# TinyML in Industrial IoT: A Systematic Review of Applications, System Components, and Methodologies

**DOI:** 10.3390/s26082550

**Published:** 2026-04-21

**Authors:** Shahad Alharthi, Muhammad Rashid, Malak Aljabri

**Affiliations:** Department of Computer and Network Engineering, College of Computing, Umm Al-Qura University, Makkah 21955, Saudi Arabia; mfelahi@uqu.edu.sa (M.R.);

**Keywords:** TinyML, industrial internet of things (IIoT), edge intelligence, machine learning, resource-constrained devices, model optimization, on-device inference

## Abstract

Tiny Machine Learning (TinyML) enables Machine Learning (ML) models to run on resource-constrained devices, which is critical for Industrial Internet of Things (IIoT) systems requiring low latency, energy efficiency, and local decision-making. Nevertheless, deploying TinyML in IIoT remains challenging due to diverse applications, hardware, frameworks, and deployment methodologies, highlighting the need for a structured and focused review. Existing review articles mainly address general IoT or edge AI, leaving a critical gap in a unified and systematic understanding of TinyML applications, system components, and methodologies within IIoT contexts. Consequently, this systematic literature review (SLR) addresses this gap by analyzing 35 peer-reviewed studies published between 2018 and 2026, offering a comprehensive and structured synthesis of TinyML-enabled IIoT systems. The selected works are synthesized across three major dimensions: applications, system components, and methodologies. In terms of applications, TinyML is primarily used for predictive maintenance, equipment monitoring, anomaly detection, energy management, and general-purpose applications. The general category captures cross-domain solutions that do not fit into a single industrial application. A comparative analysis of all application categories is conducted in terms of accuracy, latency, memory, and energy. For system components, a structured comparison shows how hardware, software, and sensing choices shape performance and applicability. Hardware platforms are grouped by microcontroller families, highlighting dominant types. Software frameworks are summarized, showing the widespread use of lightweight toolchains for on-device inference. Sensor types are categorized, with vibration sensing most common. They are supported by other sensing methods such as vision, sound (acoustic), and environmental sensors. Finally, the methodologies examined in this SLR provide a comprehensive view of the data foundations, model selection, and optimization strategies. In short, this SLR converges diverse TinyML–IIoT applications, microcontroller-based hardware, lightweight software frameworks, sensing modalities, varied datasets, and optimization strategies, while also identifying challenges and future research directions.

## 1. Introduction

The Industrial Internet of Things (IIoT) traces its origins to the mid-twentieth century, when factories adopted electronic control systems to enhance manufacturing processes [1]. The introduction of the programmable logic controller in 1968 was a significant milestone in industrial computing [2]. In the 1970s and 1980s, distributed control systems extended automation from individual machines to coordinated networks of sensors and actuators [3]. In the 1990s, the widespread use of the Internet and wireless communication technologies enabled industrial devices to connect to larger digital networks. These developments laid the foundation for new industrial integration. By the early 2010s, the term IIoT emerged, describing an industrial counterpart of the IoT in which physical and cyber systems are tightly integrated under Industry 4.0 [4,5].

Today, IIoT is a central component of digital transformation across many industrial sectors [6,7]. It allows critical infrastructures to continuously monitor equipment and collect operational data to support real-time decision-making [8]. Consequently, it enables predictive maintenance, improved energy efficiency, and reduced operational costs of industrial assets [9]. IIoT has also contributed to the development of the smart factory concept, where cyber–physical systems autonomously and adaptively optimize production lines [10]. However, large-scale IIoT deployments still face significant challenges in data security, scalability, and energy consumption, particularly in large, heterogeneous industrial networks. These challenges have encouraged the use of emerging technologies such as Tiny Machine Learning (TinyML) [11]. It is considered a subfield of artificial intelligence that focuses on deploying ML models on ultra-low-power microcontroller devices. These devices typically have less than 1 MB of RAM [12].

**Figure 1:**
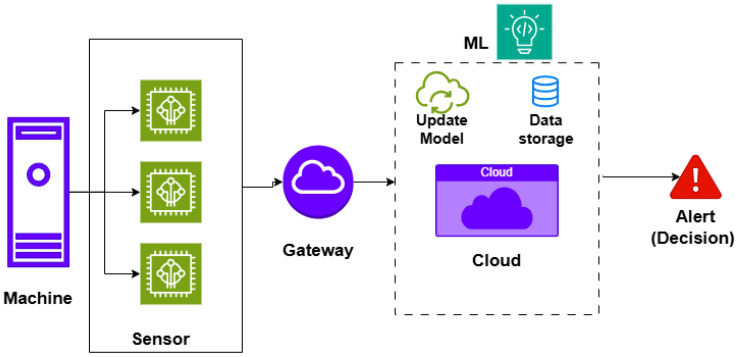
Traditional IIoT architecture based on centralized cloud-centric data processing, where sensor nodes transmit raw data to gateways and cloud servers for storage, analysis, and inference.

**Figure 2:**
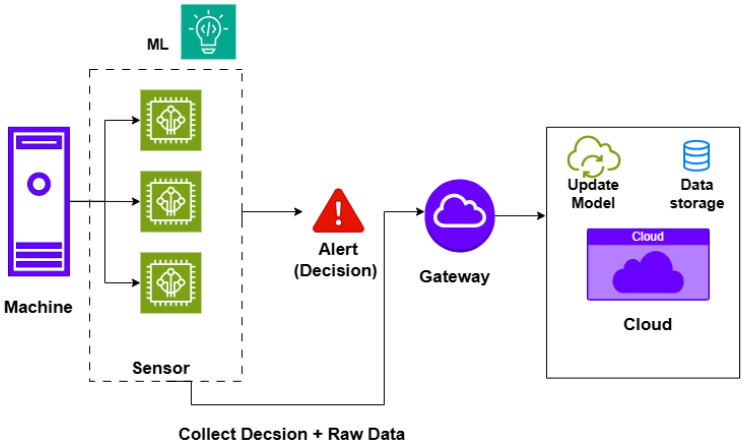
TinyML-enabled IIoT architecture incorporating distributed on-device intelligence, where ML inference is executed locally on microcontrollers.

In traditional IIoT systems (Figure 1), sensor nodes send raw data to a gateway. The gateway then forwards this data to the cloud, where it is stored and used for model training and inference. However, this architecture suffers from high communication costs, more energy consumption, and increased latency, making it less suitable for time-sensitive or bandwidth-limited scenarios. In contrast, the TinyML-enabled IIoT architecture (Figure 2) introduces a distributed intelligence layer. It enables on-device inference, allowing sensors and embedded systems to perform data analytics and make real-time decisions locally without constant reliance on cloud connectivity [13]. In other words, only periodic model updates are sent to the cloud. Offline or periodic synchronization keeps edge models accurate. This approach reduces data transmission requirements, lowers latency, and enhances privacy and energy efficiency [14].

While TinyML represents the most resource-constrained form of on-device intelligence, it exists within a broader landscape of edge-based machine learning approaches [15]. Embedded machine learning (ML) refers to ML executed on embedded systems such as microcontrollers, DSPs (Digital Signal Processors), or lightweight SoCs (Systems-on-Chips), without necessarily requiring the extreme memory and energy efficiency that characterize TinyML [16]. Edge AI employs an even wider scope, including all AI processing performed near the data source (gateways, industrial computers, or edge servers) with significantly greater computational capacity [17,18]. Within this hierarchy, TinyML is the most constrained subset of both embedded ML and edge AI, uniquely enabling real-time intelligence directly at the sensor level in IIoT environments.

In IIoT environments, TinyML has demonstrated potential in applications such as vibration analysis, predictive maintenance, quality inspection, anomaly detection, structural health monitoring, and smart energy management [19]. By embedding lightweight learning models directly into industrial sensor nodes and microcontrollers, TinyML transforms passive IIoT devices into intelligent edge agents capable of making context-aware decisions.

### 1.1. Motivation for a Systematic Literature Review on TinyML in IIoT

The integration of TinyML into IIoT systems is increasing; however, this convergence introduces significant complexity [20]. The diversity of applications, components, methodologies, and constraints and the limited availability of labeled datasets make deployment challenging. Similarly, designing TinyML models often requires advanced optimization techniques such as quantization, pruning, and knowledge distillation [21]. It also demands standardized toolchains and libraries that support portability across heterogeneous resource-constrained microcontroller architectures [22]. Despite growing interest and promising results, existing research remains fragmented across multiple domains. A systematic literature review (SLR) is therefore essential to consolidate current knowledge. It should not only identify gaps in applications, system components, model design, and deployment methodologies but also outline future research directions. Such a review can accelerate the adoption of TinyML in IIoT environments while addressing critical issues of scalability, reliability, and interoperability.

### 1.2. Overview of Existing Reviews on TinyML in IIoT

Existing reviews can be grouped into three categories: TinyML-focused reviews, ML-in-IIoT reviews, and recent surveys on TinyML and on-device edge intelligence. A summary of these surveys is provided in Table 1.

**TinyML-focused Reviews:** Early studies explored frameworks and optimization techniques for enabling ML inference on microcontrollers. They discussed methods such as model compression, quantization, and pruning [23]. While these reviews offered valuable insights into TinyML design, they did not address industrial contexts or IIoT-specific challenges such as latency and reliability. A more recent review categorized TinyML research into algorithm-oriented, hardware-oriented, and co-design approaches. However, it lacks an analysis of real-time industrial applications [24]. Another review focused on predictive maintenance, showing how on-device models can detect faults using vibration and temperature data. Still, its scope was limited to maintenance and excluded other IIoT domains such as energy optimization and quality inspection [19].

**Reviews Focused on ML in IIoT:** Several systematic reviews have analyzed the integration of ML in IIoT systems but have not explicitly considered TinyML. The work in [25] provided valuable insights into industrial data analysis; it relies on cloud or server-side processing rather than edge intelligence [25]. Similarly, the work in [26] discussed model reusability and cross-domain adaptation but did not address TinyML or energy-efficient inference on embedded devices [26]. The work in [27] examined the use of ML on IoT data, emphasizing interpretability and model transparency while overlooking the constraints of local learning and resource-limited hardware. The work in [28] also introduced a taxonomy of ML methods for smart manufacturing [28], categorizing techniques by data types and industrial objectives; however, it did not address TinyML, edge-level deployment, or power-efficient computing.

**Surveys on TinyML and Edge Intelligence:** Recently, some surveys have begun examining TinyML within broader edge-computing environments [29,30]. The work in [29] discusses embedded AI architectures, hardware platforms, and performance considerations across diverse IoT scenarios. However, the analysis remains centered on general IoT and embedded AI settings rather than addressing the specific deployment constraints of IIoT systems. More recently, the work in [30] explored the role of TinyML in industrial IoT within the transition from Industry 4.0 to Industry 5.0. Nevertheless, the work focuses primarily on conceptual perspectives, without providing a detailed synthesis of TinyML deployment architectures, datasets, or optimization techniques.

### 1.3. Research Gap

Although previous review articles and surveys provide valuable insights into TinyML techniques and ML applications in IIoT environments, their scope remains fragmented and incomplete. Several works examine TinyML in general IoT contexts [23,24,29], but they do not address the unique constraints of industrial systems. Other reviews focus on ML in industrial settings [25,26,27,28], yet these studies rely predominantly on cloud or server-side processing and overlook TinyML-based on-device intelligence. Furthermore, some surveys narrow their scope to specific use cases such as predictive maintenance [19], limiting their applicability to broader IIoT domains. Even recent works that discuss TinyML within the context of Industry 5.0 [30] emphasize conceptual perspectives rather than providing a technical synthesis of the domain. As a result, the existing literature lacks a unified and technically grounded review of TinyML specifically tailored to IIoT environments.

As summarized in Table 1, no prior review offers a comprehensive analysis of TinyML within IIoT, despite the increasing need for energy-efficient, real-time, and scalable intelligence at the industrial edge. TinyML provides a promising alternative by enabling on-device inference on ultra-low-power microcontrollers; however, the absence of a consolidated perspective obscures understanding of the hardware platforms, software frameworks, model-design strategies, and optimization techniques required for practical IIoT deployment. This systematic literature review addresses this gap by delivering the first unified synthesis of TinyML applications in industrial environments, examining deployment architectures, industrial use cases, sensing modalities, hardware constraints, and open research challenges that shape the future of TinyML-enabled IIoT systems.

### 1.4. Research Questions

To address the identified research gaps, this SLR aims to answer the following research questions (RQs):

- **RQ1:** In which industrial domains and operational tasks has TinyML been applied within IIoT systems?
- **RQ2:** What system components (hardware platforms, software frameworks, and sensor modalities) are most commonly used for deploying TinyML in IIoT?
- **RQ3:** What methodologies (datasets, model architectures, and optimization strategies) in TinyML-IIoT are employed to adapt ML models for resource-constrained IIoT devices?
- **RQ4:** How is the performance of TinyML models in IIoT applications evaluated, and what metrics are most frequently reported?
- **RQ5:** What are the key limitations and open research challenges in applying TinyML to IIoT, and what future research directions are proposed?

These questions guide the entire review process, from the selection of studies to the synthesis of results, and aim to provide a comprehensive overview of the current state and future opportunities of TinyML in IIoT.

### 1.5. Contributions

To the best of our knowledge, this study is among the first systematic literature reviews to provide a comprehensive examination of TinyML within the IIoT context. Unlike previous reviews that addressed TinyML and IIoT separately, this SLR integrates both perspectives to deliver a unified and in-depth analysis of their intersection. The main contributions are summarized as follows:

1. **Comprehensive synthesis of TinyML in IIoT:** It explicitly examines how TinyML has been adopted in IIoT systems across different domains such as predictive maintenance, quality control, anomaly detection, and smart energy management.
2. **Analysis of hardware and software ecosystems:** It identifies the most common microcontroller units (MCUs)/edge devices, software frameworks (e.g., TensorFlow Lite Micro, Edge Impulse), and sensor modalities used for deploying TinyML in IIoT.
3. **Evaluation of datasets, model architectures, and optimization techniques:** It analyzes the datasets, model types, and optimization techniques used to adapt ML algorithms for resource-constrained industrial environments, including model compression, quantization, and pruning.
4. **Performance assessment and benchmarking:** It summarizes how TinyML performance in IIoT is evaluated, highlighting frequently reported metrics such as accuracy, inference latency, and energy efficiency.
5. **Identification of challenges and future research directions:** It highlights the technical and methodological limitations reported in the literature, including deployment complexity, scalability, and lack of standardized benchmarking, and outlines promising future research directions.

Through these contributions, this SLR provides an integrated foundation for researchers and practitioners aiming to design, optimize, and deploy TinyML-based intelligent systems in industrial IoT applications.

### 1.6. Layout of the SLR

This SLR is organized into well-defined sections that follow the adopted methodological workflow. The introduction is followed by the research methodology (Section 2), which outlines the categories and details the review protocol for selection of studies. The selected studies are then classified according to the research problems they address (Section 3), followed by a system-component taxonomy covering hardware, frameworks, and sensors (Section 4). Next, the review examines methodologies related to datasets, model architectures, and optimization strategies (Section 5). Subsequently, the study summarizes key challenges and future research directions (Section 6). Based on the contents of Section 3, Section 4, Section 5 and Section 6, answers to the formulated research questions are provided, along with the limitations of this SLR (Section 7). Finally, the article concludes with Section 8.

## 2. Research Methodology

This SLR adopts established systematic review guidelines to ensure a transparent and reproducible methodology. Section 2.1 begins by defining the background categories that structure the analysis, followed by a detailed review protocol in Section 2.2. The protocol outlines the study selection criteria, search strategy, and data extraction procedures. These subsections collectively describe how relevant studies were identified, screened, and synthesized to address the research questions.

### 2.1. Background on Categories

The selected research articles are organized into three complementary classification types that reflect different analytical perspectives: (1) The problem-driven classification groups studies according to the primary industrial challenge they address, enabling an application-level understanding of how TinyML is used in IIoT contexts. (2) The system component taxonomy groups studies by the hardware platforms, software frameworks, and sensor modalities, highlighting the core technological elements. (3) The methodology classification focuses on datasets, model architectures, and optimization strategies, capturing how researchers adapt ML techniques to meet the constraints of IIoT systems. Together, these three classification types provide a structured and multidimensional view of TinyML-IIoT research.

#### 2.1.1. Problem-Driven Classification

Classifying studies by their dominant research problem is common in systematic reviews, particularly in multidisciplinary fields such as TinyML-enabled IIoT systems. This classification organizes diverse contributions and offers an application-level view of TinyML in industrial challenges. By focusing on each study’s primary objective, this category clarifies the industrial contexts of TinyML solutions [11]. In other words, problem-driven classification emphasizes the main system objective and the industrial environment rather than the specific algorithms, model architectures, or hardware platforms employed. These technical aspects are analyzed separately [31].

Selected studies are grouped into five problem-oriented categories: Smart Manufacturing and Predictive Maintenance, Industrial Equipment and Condition Monitoring, Smart Energy Management, Quality Control and Anomaly Detection in Production Lines, and General Applications. Although these categories are defined separately, overlap between them is expected in real industrial systems, as a single TinyML solution may address multiple operational objectives. Consequently, some studies may appear in more than one category when their contributions span multiple industrial domains, reflecting the practical nature of IIoT environments where TinyML solutions often support several operational goals simultaneously. This problem-oriented classification highlights TinyML’s flexibility across different IIoT application domains and establishes a consistent basis for comparative analysis in the remainder of the review. It is important to note that the reviewed studies differ in their level of implementation, ranging from simulation-based evaluations to laboratory prototypes, MCU-level deployments, and industrially validated systems.

#### 2.1.2. SystemComponent Taxonomy: Hardware, Frameworks, and Sensors

**Figure 3:**
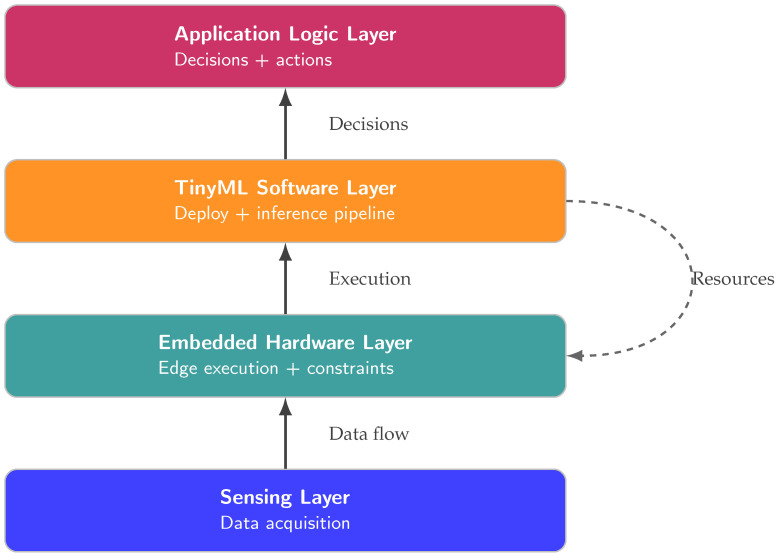
Structural layers of TinyML in Industrial IoT, from sensor data acquisition to application- level decision making.

This category presents a structural taxonomy of TinyML system components in IIoT environments. It emphasizes three interrelated dimensions: hardware processing platforms, software frameworks/libraries, and sensing modalities. These dimensions constitute the fundamental technical building blocks shared by most TinyML-IIoT systems. It enables a unified description of the literature despite differences in application domains [32], as illustrated in Figure 3.

As illustrated in Figure 3, the TinyML-IIoT layered architecture begins with the Sensing Layer, which acquires data from the industrial physical environment [33]. This layer serves as the primary source of input data and strongly influences subsequent system design choices. It is followed by the Embedded Hardware Layer, which provides the execution environment for TinyML models at the industrial edge and imposes constraints related to memory capacity, energy consumption, and connectivity [34,35]. Above the hardware layer lies the TinyML Software Layer, encompassing model deployment and on-device inference pipelines [36]. This layer acts as an intermediary between raw sensor data and higher-level system behavior. Finally, the architecture includes an optional Application Logic Layer that uses the outputs of TinyML models to support industrial decisions and actions, depending on the system’s nature.

The layered organization of Figure 3 reflects the principle that sensing choices determine the characteristics of input data, hardware platforms define execution constraints, and software frameworks govern how models are built, deployed, and executed on resource-constrained devices. Accordingly, this section provides a methodological overview of these layers through structured classification tables: a hardware taxonomy that groups platforms by architectural families, a software taxonomy that describes development and deployment environments, and a sensor taxonomy that categorizes measurements by the type of physical phenomenon observed. This framework establishes a consistent basis for the detailed classification presented in the following subsections, without introducing performance analysis at this stage [37].

#### 2.1.3. Methodologies: Datasets, Model Architectures, and Optimization Strategies

This category focuses on three interrelated technical dimensions: datasets, model architectures, and optimization techniques.

- First, the dataset dimension addresses the types of data sources used for training and evaluation. Datasets are organized into two categories: (1) operational and real-world sensor datasets, which reflect actual operating conditions in industrial or experimental environments, and (2) public, benchmark, and synthetic datasets, which include standardized datasets and simulated data used to support reproducible experimentation [33,38]. This classification clarifies the context in which models are trained and tested, without evaluating data quality or outcomes.
- Second, this category introduces a classification of model architectures used in TinyML systems. This dimension focuses on the structural design of the models, which determines how input data are processed on embedded devices. It captures architectures suitable for different data representations, such as image data, time-series signals, and low-dimensional sensor measurements [34].
- Third, this category describes optimization techniques. Optimization is considered a set of strategies that may target the model structure, numerical representation, execution behavior, or learning process. These techniques are organized into clearly defined categories based on how they are reported in the reviewed literature, supporting a structured and reproducible presentation [37].

### 2.2. Review Protocol Development

Developing a structured and well-defined review protocol is a fundamental step in conducting a systematic literature review. This article adopts a systematic literature review approach aligned with the PRISMA (Preferred Reporting Items for Systematic Reviews and Meta-Analyses) guidelines. It ensures a transparent and reproducible review process [39]. It comprises three main stages: defining selection and exclusion criteria, conducting the search process, and performing data extraction and synthesis. Similar methodological approaches have been successfully applied in prior reviews on AI methods in medical imaging [40], underwater acoustic communication [41], bridge health monitoring [42] and embedded systems development [43], demonstrating the robustness and adaptability of this framework across diverse domains.

#### 2.2.1. Selection and Rejection Criteria

To ensure the inclusion of high-quality, relevant studies, a set of inclusion and exclusion criteria was established before the search. Studies were selected based on the following criteria:

- **Subject relevance:** Studies must explicitly address TinyML or on-device machine learning within an industrial or IIoT context, and provide evidence relevant to at least one of the research questions (applications, system components, methodologies, performance, or challenges).
- **Alignment with classification categories:** Each study must contribute to at least one of the three dimensions defined in the background on categories: (i) problem-driven classification, (ii) system components (hardware, frameworks, sensors), or (iii) methodologies (datasets, model architectures, optimization strategies).
- **Publication period:** Only studies published between 2018 and 2026 were included to capture the emergence and evolution of TinyML in IIoT. Earlier works were excluded unless foundational to TinyML or embedded ML.
- **Publication source:** Only peer-reviewed journal articles and conference papers indexed in IEEE Xplore, Elsevier (ScienceDirect), SpringerLink, and the ACM Digital Library were considered to ensure scientific rigor.
- **Industrial relevance:** Included studies must demonstrate a clear connection between TinyML techniques and industrial or IIoT applications.
- **Results-oriented evidence:** Studies must report experimental results, quantitative evaluations, prototypes, or real-world/industrial testbeds. Conceptual, theoretical, or purely survey-based works without empirical validation were excluded.
- **Redundancy:** Duplicate or substantially overlapping publications were removed, with preference given to the most complete, updated, or extended version of the study.

#### 2.2.2. Search Process

A systematic literature search was conducted across four major scientific databases: IEEE Xplore, Elsevier (ScienceDirect), SpringerLink, and the ACM Digital Library. The literature search was conducted between 17 December 2025 and 16 March 2026. The same core Boolean search string was used across all selected databases to ensure consistency. The search and screening process followed the standard stages of systematic literature reviews, including identification, screening, eligibility assessment, and final inclusion. To ensure both breadth and precision, the search strategy combined TinyML-related terms with IIoT-specific keywords using Boolean operators. The search was limited to studies published between 2018 and 2026 to capture recent developments in the field. The primary Boolean query used in this review is defined as follows:

(“TinyML” OR “Tiny Machine Learning” OR “on-device machine learning” OR “micro ML” OR “embedded machine learning” OR “low-power ML”)

AND

(“Industrial Internet of Things” OR “IIoT” OR “industrial IoT” OR “Industry 4.0” OR “smart factory” OR “connected industrial devices”)

To validate search coverage and to better understand the terminology commonly used in the literature, both individual and combined keyword queries were initially explored across the selected databases. Table 2 summarizes the number of records retrieved for each keyword and keyword combination. These counts are reported for exploratory purposes and are not cumulative, as overlapping records frequently appear across multiple queries and databases. The results reveal that broad and generic terms (e.g., *Edge AI*, *Low-power ML*, or *Industry 4.0*) yield huge numbers of records, many of which fall outside the scope of TinyML-based IIoT research. Consequently, more restrictive Boolean combinations were intentionally adopted to reduce noise and improve the relevance of results. Although the combined queries yield fewer records, they provide a more focused, domain-specific dataset suitable for systematic screening and qualitative synthesis.

**Figure 4:**
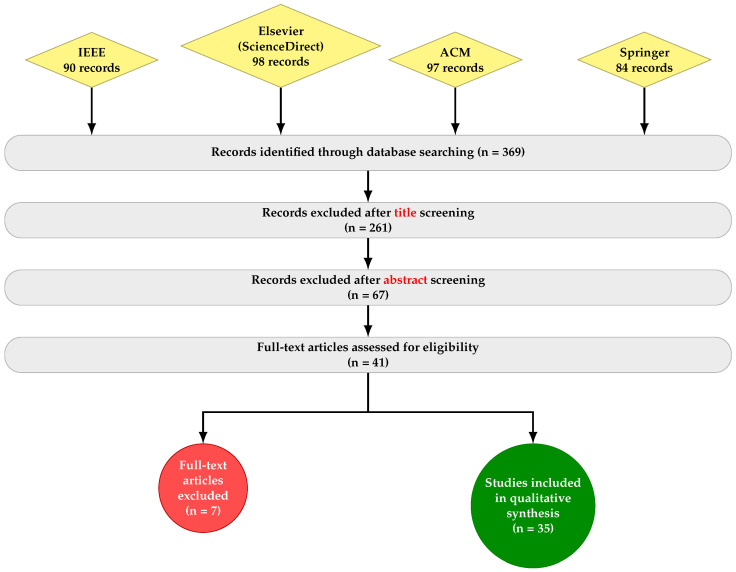
PRISMA-based study selection flow diagram illustrating the identification, screening, eligibility assessment, and final inclusion of studies (2018–2026).

The initial database search yielded 369 records. A title-based screening was first applied to remove clearly irrelevant studies, reducing the number of records to 108. Subsequently, abstract screening was conducted, resulting in 41 studies retained for full-text assessment. After applying the eligibility criteria via full-text review, 7 studies were excluded for duplication, insufficient experimental validation, or limited industrial relevance. Consequently, a final set of 35 studies was retained for qualitative synthesis and detailed analysis. Although the final number of selected studies may appear limited, this reflects the strict inclusion criteria applied in this review as well as the emerging nature of TinyML applications in industrial IoT environments. The overall study selection process is illustrated in Figure 4.

#### 2.2.3. Data Extraction and Synthesis

After selecting the final set of studies, a structured data extraction and synthesis process was applied to ensure consistency and traceability across the review. Key information was systematically extracted from each study, including the IIoT domain and application context; experimental performance indicators; hardware platforms; software frameworks; sensing modalities; datasets; model architectures; and optimization techniques. The extracted data were organized into thematic synthesis dimensions for comparative analysis, as summarized in Table 3. Studies were classified by dominant IIoT domains (Table 4), while performance attributes such as accuracy/F1-score, latency, memory, and energy consumption were synthesized in application-specific tables (Table 5, Table 6, Table 7 and Table 8).

**Table 3:** Overview of data synthesis dimensions and corresponding result tables.

Description	Synthesized Results (Tables)
IIoT domain classification	Table 4: TinyML–IIoT studies classified by industrial domain and application.
Performance evaluation	Table 5, Table 6, Table 7, Table 8 and Table 9: Performance results of TinyML–IIoT applications across accuracy, latency, memory, and energy.
Hardware platforms	Table 10: Hardware platforms grouped by microcontroller family, processor architecture, integrated accelerators, and connectivity features.
Software frameworks	Table 11: Software frameworks and platforms in TinyML–IIoT studies.
Sensor modalities and properties	Table 12: Sensor types in TinyML–IIoT and their measured properties.
Datasets	Table 13: Operational and real-world sensor datasets used in TinyML–IIoT studies. Table 14: Public, benchmark, and synthetic datasets used for TinyML evaluation.
Model architectures	Table 15: Summary of model architectures used in the reviewed TinyML–IIoT studies.
Optimization techniques	Table 16: Categories of optimization techniques applied in TinyML–IIoT systems.
Additional synthesis dimension.	Table 17: Summary mapping of data types, model families, and typical optimization techniques (synthesized from Table 13, Table 14, Table 15 and Table 16).

**Table 4:** Classification of Selected TinyML–IIoT Studies According to the Dominant Research Problem.

Refs.	IIoT Domain	Number of Articles
[34,44,45,46,47,48]	Smart Manufacturing & Predictive Maintenance	6
[45,47,49,50]	Industrial Equipment/Condition Monitoring	4
[51,52,53,54]	Smart Energy Management	4
[46,55,56,57,58,59]	Quality Control & Anomaly Detection	6
[33,36,37,38,60,61,62,63,64,65,66,67,68,69,70,71,72,73]	General Applications	18

**Table 5:** Comparative experimental results in smart manufacturing and predictive maintenance.

Ref.	Accuracy/F1 (%)	Memory Flash/RAM (KB)	Latency (ms)	Energy per Inference
[44]	99.0	512/64	–	Self-powered (piezoelectric harvester)
[34]	– (RUL: RMSE/score)	2048/512	1.81	–
[45]	F1-normal: 91.3/F1-abnormal: 67.9	1024/256	–	13.3–20.6 μJ/inference
[46]	F1-normal: 87.6/F1-states: 96.8	1024/256	0.106	1.16 µJ/inference
[47]	99.36	1024/256	30	–
[48]	96.4	**–**	–	–

**Table 6:** Comparative experimental results in Industrial Equipment/Condition Monitoring.

Ref.	Accuracy/F1 (%)	Memory Flash/RAM (KB)	Latency (ms)	Energy per Inference
[47]	99.36	1024/256	30	–
[49]	96.5 (F1: 96.64)	8192/512	25	–
[45]	F1-normal: 91.3/F1-abnormal: 67.9	1024/256	–	13.3–20.6 μJ/inference
[50]	–	1024/256	–	–

**Table 7:** Comparative experimental results in Smart Energy Management studies.

Ref.	Result/Contribution	Memory Flash/RAM (KB)	Latency (ms)	Energy Metric
[51]	SoC error metrics	1024/256	–	–
[52]	Energy efficiency improvement: 38%	512/64	–	–
[53]	Energy profiling (TinyEP)	2048/264	0.1	–
[54]	Energy reduction: 29%; Compression gain: 18%	2048/786	–	–

**Table 8:** Comparative results for Quality Control and Anomaly Detection in Production Lines.

Ref.	Accuracy/F1 (%)	Memory Flash/RAM (KB)	Latency (ms)	Energy per Inference
[46]	F1-normal: 87.6/F1-states: 96.8	–/–	0.106	1.16 μJ/inference
[55]	99/98	–/–	240/233	192/186 mJ
[56]	100/99.8/100	8.1/0.8	1.16	–
[57]	98	–/–	–	–
[58]	–	–/–	–	–
[59]	F1: 97.46% (TinyFL), 96.92% (TTFL)	16	16,490	4.28

**Table 9:** General TinyML applications in IIoT: types, roles, relevance, and best reported results.

Ref.	Contribution Type	Role of TinyML	IIoT Relevance	Best Results
[60]	Model management & on-device learning	Online, meta-learning, adaptive edge models	Useful for dynamic and evolving industrial environments	Up to +12% accuracy improvement
[36]	TinyML deployment workflow	Quantization, deployment pipeline for MCUs	Applicable to any MCU-based IIoT deployment	ESP-FOMO (60–70% accuracy)
[61]	Framework comparison	Evaluation of TinyML toolchains	Helps select suitable tools for IIoT systems	–
[62]	Embedded AI benchmarking	Performance evaluation of TinyML tools	Supports IIoT integrators in toolchain selection	–
[63]	Distributed TinyML learning	Model splitting across multiple edge nodes	Ideal for large industrial sensor networks	89% latency reduction
[64]	Federated learning (FL + TL)	On-device training with privacy preservation	Useful for decentralized IIoT systems	86.48% accuracy
[65]	FL over LoRa mesh networks	Low-power, low-bandwidth collaborative learning	Suitable for remote and bandwidth-limited IIoT	–
[66]	Distributed TinyML on sensor networks	Model partitioning & distributed execution	Useful for dense IIoT sensing with strict memory limits	95.5% validation accuracy
[67]	Continual TinyML learning	Quantized latent replay	Adapts to changing IIoT operating conditions	–
[33]	General-purpose vibration classification	Running-state recognition on the MCU	Transferable to industrial mobile assets	>99% accuracy
[38]	TinyML-based data compression	Edge compression of vibration signals	Reduces wireless load in SHM & other IIoT systems	99.96% compression (TAC)
[68]	Impact localization (SHM)	Lightweight vibration classification on the MCU	Applicable to structural monitoring in IIoT	98.71% accuracy (Random Forest)
[69]	TinyML-based vision system	Embedded visual landing guidance	Transferable to industrial robotics & drones	–
[70]	Parallelized classical ML	Ultra-efficient non-neural ML inference	Suitable for battery-powered IIoT sensors	3.7 μJ per inference
[71]	Processing-in-sensor architecture	Ternary MLP executed directly in the sensor frontend	Reduces communication in IIoT sensing layers	–
[37]	Approximate TinyML kernels	Energy-efficient CNN kernels	Useful for industrial vision workloads	72.4% (approximate AlexNet)
[72]	TinyML anomaly detection for IoT devices	Real-time threat/anomaly detection at the edge	Supports IIoT safety and device protection	–
[73]	Memory-efficient anomaly detection	Low-memory time-series inference on MCU	Applicable to IIoT anomaly detection pipelines	2–7× memory reduction; <0.1 s inference latency

System-level characteristics were consolidated across hardware, software, and sensing layers (Table 10, Table 11 and Table 12). Dataset types—including real-world, public, and synthetic sources—are summarized in Table 13 and Table 14, while model architectures and optimization strategies are captured in Table 15 and Table 16. Additionally, Table 17 provides a synthesized mapping of data types, model families, and typical optimization techniques, integrating insights from Table 13, Table 14, Table 15 and Table 16 to highlight methodological patterns across TinyML–IIoT studies. Given the heterogeneity of applications and evaluation metrics, a qualitative synthesis using structured tables was adopted to highlight dominant trends and research gaps.

**Table 10:** Hardware platforms grouped by microcontroller family.

Refs.	Device/Board	CPU/Architecture	Integrated Accelerators	Connectivity
**nRF52 Family (BLE-based MCUs)**				
[44]	nRF52832	ARM Cortex M4, 64 MHz	FPU	BLE, GPIO
[50]	Arduino Nano 33 BLE Sense	Cortex M4F @ 64 MHz	FPU	BLE
[68]	Arduino Nano 33 BLE Sense	Cortex M4F @ 64 MHz	FPU	BLE
[60]	Arduino Nano 33 BLE Sense	Cortex M4F @ 64 MHz	FPU	BLE
[63]	nRF52840 board	Cortex M4 @ 64 MHz	–	BLE
[58]	Custom nRF52832 + BHI260AP	Cortex M4 @ 64 MHz + sensor hub	Sensor fusion engine	BLE, NFC
[46]	Arduino Nano 33 BLE Sense; RP2040	Cortex M4 @ 64 MHz; Cortex M0+, 133 MHz	FPU (nRF52840)	BLE, USB
[45]	Arduino Nano 33 BLE Sense; Raspberry Pi Pico	Cortex M4 @ 64 MHz; Cortex M0+, 133 MHz	FPU (nRF52840)	BLE, USB
**STM32 Family**				
[34]	STM32F767ZI	Cortex M7 @ 216 MHz	FPU	USB
[37]	STM32U575ZI Q	Cortex M33, 160 MHz	–	UART, SPI, I^2^C, USB, GPIO
[38]	STM32 (LoRa node)	Cortex M4 up to 80 MHz	–	LoRaWAN
[61]	STM32F401RE; STM32H743; Raspberry Pi 4	Cortex M4; Cortex M7; Cortex A72	–	–
[62]	STM32 NUCLEO L4R5ZI; Renesas RX65N	Cortex M4; RXv2	–	–
**ESP32/ESP8266 Family**				
[33]	ESP32	Xtensa dual core @ 240 MHz	–	Wi-Fi
[66]	ESP32 S3	Xtensa LX7, dual core	–	Wi-Fi, BLE, GPIO
[36]	ESP32 CAM	Xtensa 32 bit @ 240 MHz	–	Wi-Fi, Bluetooth
[49]	Seeed XIAO ESP32S3	Xtensa LX7, dual core	AI accelerator	Wi Fi, BLE, MQTT
[73]	ESP8266/ESP32	Generic MCU	–	–
[59]	ESP32-S3	Xtensa LX7 dual-core MCU	–	LoRaWAN
**Accelerator-Based and Vision-Oriented Platforms**				
[55,69]	OpenMV Cam H7 Plus	Cortex M7 @ 480 MHz	–	Camera, USB, UART, SPI
[70]	GAPUINO (GAP8)	RISC V (PULP)	ART accelerator, DSP, FPU	–
[70]	VCU118 FPGA	FPGA based	Hardware acceleration	–
[56]	Wio Terminal	Cortex M4F (ATSAMD51) @ 120 MHz	–	Wi-Fi, BLE, LCD, GPIO, USB
**Multi-board/Mixed-Hardware Platforms**				
[53]	Raspberry Pi Pico W	Dual core Cortex M0+ @ 240 MHz	–	Wi Fi, BLE, ADC, GPIO
[57]	Nano 33 BLE Sense; Portenta H7	Cortex M4F; Cortex M7 + Cortex M4	–	BLE; Wi-Fi, BLE, USB
[51]	Arduino Nano 33 BLE Sense; ESP32 S3	Cortex M4; Xtensa @ 240 MHz	–	BLE; Wi Fi, BLE, USB
[64]	Arduino WiFi Rev2; ESP8266; ESP32; MKR1010; RPi Zero W; RPi 3B+	Cortex M0+/M4; Xtensa; ARM1176; Cortex A53	–	Wi-Fi, BLE, USB
**Heterogeneous and Multi-Processor Platforms**				
[67]	VEGA SoC + STM32L476RG	RISC V multi core + Cortex M4	Parallel ML accelerator	SPI, UART, I^2^C
[65]	Portenta H7; TTGO LoRa32	Cortex M7/M4; Xtensa LX6	–	LoRa, UART
**Benchmarking/Simulation/Hardware Not Specified**				
[47,48,52,54,71,72]	–	–	–	–

**Table 11:** Summary of software frameworks and platforms used in TinyML IIoT studies.

Refs.	Software Framework/Platform	Total Articles
[38,46,52,53,59,60,62,63,64,66,67,70,73]	Custom Proprietary Frameworks	13
[33,34,36,44,51,55,56,57,61,62,69]	TensorFlow Lite Micro (TFLM)	11
[45,47,50,60,63,64,65,68,70,73]	Direct On Device Implementation	10
[33,36,49,57]	Edge Impulse Platform	4
[54,58,71,72]	Simulation-Based	4
[37,53,62]	ARM CMSIS NN/DSP Libraries	3
[34,62]	STM X CUBE AI Toolchain	2
[46]	Imagimob Studio	1
[61]	Larq/QKeras Frameworks	1

**Table 12:** Summary of sensor types used in the reviewed TinyML IIoT studies.

Refs.	Sensor Type	Measured Property
[33,38,44,47,49,50,58,68,73]	Motion and Vibration Sensors (Micro-Electro-Mechanical Systems (MEMS))	Accelerometer, gyroscope, IMU, piezoelectric sensors
[46,59]	Pressure and Flow Sensors	Hydraulic pressure sensors, differential pressure sensors
[50,56,66]	Environmental and Chemical Sensors	Gas sensors, temperature, humidity, environmental sensing
[55,57,69]	Vision and Imaging Sensors	RGB cameras, IR cameras
[51,53]	Energy Measurement Sensors	Voltage and current sensing
[48,65]	Acoustic and Audio Sensors	Microphones, audio waveforms

**Table 13:** Operational and real-world sensor datasets used in TinyML IIoT studies.

Ref.	Dataset Type	Source/Origin	Dataset Size
[44]	Vibration signals (piezoelectric)	Custom-collected	Not specified
[45]	Duty cycle operational data	Industrial dataset	Not specified
[50]	IMU and temperature signals	Custom pump testbed	Not specified
[57]	IR thermography images	Custom PV dataset	2000 images
[55]	Industrial product images	Custom industrial dataset	Not specified
[33]	MEMS accelerometer signals	Collected from rail vehicles	Not specified
[38]	Bridge vibration and strain data	Real-world bridge monitoring	Not specified
[46]	Hydraulic pressure and motor speed	Industrial system logs	170,000 samples; 603 cycles
[56]	Gas sensor VOC responses	Custom-collected	2334 samples
[69]	RGB landing pad images	Custom aerial dataset	13,576 positive; 12,807 negative
[68]	Piezoelectric impact signals	Custom dataset	771 instances; 5000 samples each
[49]	Motor vibration (IMU)	Custom-collected	Not specified
[53]	Energy profiling traces	Custom-collected	Not specified
[51]	Battery voltage, current, and temperature	Custom-collected	Not specified
[48]	Acoustic emission ultrasonic signals	Laboratory experiment (turbine blade)	Not specified
[58]	IMU motion data	Custom human subject dataset	Not specified
[73]	Industrial IoT time series	Real industrial datasets	Not specified
[66]	Environmental IoT sensor readings	Custom ESP32 network	Not specified
[59]	IIoT machinery telemetry	CAN bus (SAE J1939)	12-dimensional feature vector

**Table 14:** Public, benchmark, and synthetic datasets used in TinyML IIoT studies.

Ref.	Dataset Type	Source/Origin	Dataset Size
[54]	Simulated IIoT process data	Synthetic simulation	Not specified
[34]	Degradation/RUL time series	Public NASA C MAPSS	4 subsets
[47]	Bearing vibration windows	Public Afshar dataset	10,000 sample windows
[70]	Speech, image, and sound benchmarks	MLPerf Tiny (Speech Commands, CIFAR 10, ToyADMOS)	105 k; 60 k; 7 k
[71]	Digit, fashion, and face images	MNIST, Fashion MNIST, CBCL Face	70 k; 70 k; unspecified
[60]	Character, audio, and presence data	Omniglot, Speech Commands, Siemens	Omniglot: 1623 classes
[63]	Time series classification	UCR Archive	Not specified
[64]	ECG, mobility, and operational signals	MIT BIH, PTB, Car Trips, DeepEST	DeepEST: 45,500 samples
[72]	IoT telemetry data	ToN IoT benchmark	23,500 instances
[61]	Activity and vibration datasets	PAMAP2, SHL, CWRU	SHL: 750 h
[65]	Keyword spotting (KWS) audio	Public GitHub dataset	480 audio samples
[67]	Image and KWS datasets	CIFAR 10, Speech Commands	Not specified
[37]	Image classification	Public CIFAR 10 dataset	60,000 images
[62]	Mixed benchmarks (image, audio, anomaly)	MNIST, CIFAR 10/100, VWW, ToyADMOS, etc.	Standard sizes
[52]	Task scheduling and energy logs	Synthetic simulation	Not specified

**Table 15:** Summary of Model Architectures Used in Reviewed TinyML–IIoT Studies.

Refs.	Model Structure	No. of Articles
[34,36,37,47,49,55,57,61,69]	CNN	12
[38,44,45,46,50,58,63,68,70,72,73]	Classical ML	9
[33,53,56,59,60,64,65,66,71]	MLP/ANN	9
[48,54,60,63,67,71,72]	Hybrid ML	7
[51]	RNN/LSTM	2
[52]	Adaptive RL	1

**Table 16:** Categories of optimization techniques used in TinyML IIoT studies.

Refs.	Technique Category	Description
[34,47,49,55,56]	Post Training Quantization	Reduces numerical precision to decrease model size, memory usage, and inference latency.
[34,49,55]	Pruning/Weight Reduction	Removes redundant parameters or factors from network matrices to reduce computational cost.
[37]	Approximate Computing	Replaces exact convolution operations with approximate MAC operations to lower energy consumption and latency.
[59,60]	Meta Learning/Online Optimization	Improves learning efficiency, reduces communication overhead, and enables adaptation under resource constraints.
[52]	Reinforcement Learning Optimization	Adaptive scheduling mechanisms to reduce latency and improve energy efficiency.
[54]	Metaheuristic Optimization	Global optimization of routing and compression strategies for energy-efficient IIoT networks.

**Table 17:** Summary mapping of data types, model families, and typical optimization techniques (synthesized from Table 13, Table 14, Table 15 and Table 16).

Data Type	Typical Model Families	Common Optimization Techniques
Vibration and acoustic signals	CNNs (1D/2D), Autoencoders	Quantization (8-bit), pruning, depthwise separable convolutions
Environmental and energy sensor data	MLPs, Classical ML (SVM, k-NN, RF)	Feature reduction (PCA, manual selection), fixed-point arithmetic, lightweight feature extraction
Sequential time-series (motor current, pressure, vibration sequences)	RNN, LSTM, GRU	Sequence truncation, recurrent kernel quantization, weight sharing

## 3. Problem-Driven Classification of TinyML-IIoT Research

We aim to categorize the selected studies based on the research problem or contribution they address within the context of TinyML-IIoT environments. This categorization is significant because it facilitates understanding prevailing research trends and identifies the industrial sectors where TinyML applications are concentrated. In doing so, this process helps create a clear research map that illustrates the spread of these technologies across different industrial domains and application scenarios. This analysis concentrates on the primary objective of the proposed system or the industrial environment in which it operates, rather than on the specific algorithms or hardware platforms employed. Accordingly, the studies are grouped into five problem-oriented categories that reflect broader trends in the use of TinyML in intelligent industrial systems (Table 4).

- **Smart Manufacturing & Predictive Maintenance**: Address TinyML-based systems designed to predict equipment failures or estimate the Remaining Useful Life (RUL), including approaches based on vibration analysis, motor condition monitoring, and the early detection of failure indicators.
- **Industrial Equipment/Condition Monitoring**: Continuous monitoring of the physical or operational state of equipment using smart sensors and embedded TinyML models to detect deviations or abnormal conditions before failures occur.
- **Smart Energy Management**: Improving energy efficiency via load scheduling and optimizing low-power device operation, using on-device TinyML for real-time processing of consumption/generation signals.
- **Quality Control & Anomaly Detection in Production Lines**: Applying TinyML for detecting visual/operational defects during manufacturing, including edge computer vision to enhance quality and reduce errors.
- **General Application**: Explore more generic TinyML-based IIoT contributions that are not tightly bound to a single, clearly defined industrial use case, such as cross-domain optimization frameworks, architecture-level enhancements, or broadly applicable monitoring and analysis solutions that can be instantiated in multiple industrial scenarios.

Although these categories are defined as distinct in terms of their primary industrial objectives, some overlap is expected in practice. For example, a TinyML solution developed for predictive maintenance may also enhance product quality or reduce energy consumption, while a generic framework under the “General Applications” category may be deployed across manufacturing, energy, or condition-monitoring scenarios. In this review, each study is assigned to the category that best reflects its dominant research problem, although studies may appear in more than one category when their contributions span multiple industrial objectives. This problem-oriented classification underscores TinyML’s versatility across diverse IIoT environments and provides a consistent basis for comparing the effectiveness of proposed solutions within and across application domains. The dominant category for each study is determined by its primary system objective and the performance metrics emphasized in the evaluation, ensuring a transparent and reproducible classification process.

### 3.1. Smart Manufacturing and Predictive Maintenance

Applications in this category address core maintenance and reliability challenges in smart industrial environments by embedding lightweight learning models directly into monitoring and diagnostic workflows (Table 5). The summarized results highlight the diversity of TinyML-based predictive maintenance solutions across domains, with performance reported in terms of accuracy/F1 scores, memory footprints, inference latency, and energy consumption.

It is important to note that the metrics reported across the reviewed studies are not directly comparable due to differences in datasets, hardware platforms, sensing modalities, and evaluation protocols. The tables presented in this section are therefore intended to provide multi-dimensional insights rather than uniform benchmarking. Their purpose is to highlight patterns, trends, and methodological diversity across TinyML-IIoT research, rather than to rank or quantitatively compare studies.

Across the evaluated TinyML studies, ref. [47] achieves the highest accuracy at 99.36%, closely followed by [44] at 99.0%, while ref. [48] reports a strong 96.4% despite not deploying on embedded hardware. In terms of memory footprint, ref. [44] is the most lightweight solution with only 512 KB Flash and 64 KB RAM, whereas ref. [34] requires the largest resources at 2048 KB Flash and 512 KB RAM. The fastest inference is achieved by [46] with a latency of just 0.106 ms, and the same study also demonstrates the highest energy efficiency at 1.16 μJ per inference, outperforming [45], which consumes 13.3–20.6 μJ. Overall, the results highlight a trade-off between accuracy, memory usage, and efficiency, with different studies excelling in different performance dimensions.

Although several studies in Table 5 do not report all performance metrics, the table remains highly informative for comparative analysis. It enables a clear comparison of accuracy, memory footprint, latency, and energy efficiency where such data are provided. At the same time, the blank entries highlight inconsistencies in reporting practices within the TinyML–IIoT literature, revealing gaps that limit full cross-study benchmarking. This contrast is itself valuable, as it underscores the need for more standardized evaluation metrics and transparent reporting in future TinyML deployments. Thus, even with partial data, the table offers a structured overview of current evidence while identifying areas where the field requires greater methodological uniformity.

Overall, Table 5 illustrates that TinyML-enabled predictive maintenance systems can deliver high accuracy and F1 performance across diverse industrial domains while maintaining strict constraints on memory, latency, and energy. These results collectively demonstrate the adaptability of TinyML for embedding intelligence directly into IIoT monitoring workflows, enabling reliable, efficient, and real-time decision support in smart manufacturing and industrial maintenance.

### 3.2. Industrial Equipment/Condition Monitoring

Industrial equipment monitoring and condition assessment represent a central application domain for TinyML within IIoT environments, driven by the need for early fault detection, reduced unplanned downtime, and improved operational reliability. Table 6 summarizes representative studies, highlighting accuracy and F1 performance, memory footprints, inference latency, and energy consumption across diverse equipment types.

Across the reviewed studies in industrial equipment and condition monitoring (Table 6), the work in [47] achieves the highest accuracy at 99.36% while operating with the smallest memory footprint (69.7 KB Flash and 29.3 KB RAM), demonstrating an efficient balance between performance and resource usage. The work in [49] also reports strong performance with an F1 score of 96.64%, though on a significantly more capable platform (8192 KB Flash and 512 KB RAM), reflecting a trade-off between accuracy and hardware cost. The work of [45] shows moderate accuracy (91.3% for normal and 67.9% for abnormal classes) but provides detailed energy measurements, consuming 13.3–20.6 μJ per inference on a 1024/256 KB device. In contrast, ref. [50] does not report accuracy, latency, or energy metrics, though it employs a typical TinyML-capable platform with 1024 KB Flash and 256 KB RAM. Overall, the studies highlight diverse design choices, with some prioritizing ultra-low memory usage, others maximizing accuracy through more capable hardware, and only a few providing energy or latency benchmarks.

### 3.3. Smart Energy Management

Smart Energy Management represents a critical domain within IIoT where TinyML techniques can enhance energy efficiency, reduce operational overhead, and enable real-time decision-making on resource-constrained edge devices. Table 7 summarizes representative studies, highlighting both direct TinyML applications and indirect contributions to energy-aware operation.

Across the smart energy management studies (Table 7), the quantitative results reveal substantial variation in hardware capabilities and optimization goals. The work in [51] employs a mid-range TinyML platform with 1024 KB Flash and 256 KB RAM to evaluate state-of-charge error metrics, whereas ref. [52] achieves a 38% improvement in energy efficiency using the smallest memory footprint in this group (512/64 KB), highlighting the feasibility of lightweight deployments. In contrast, ref. [53] utilizes a more capable 2048/264 KB device to support high-resolution energy profiling and reports the lowest latency at 0.1 ms. Ref. [54] operates on the most resource-rich platform (2048 KB Flash and 786 KB RAM) to achieve a 29% reduction in energy consumption and an 18% compression gain.

Notably, most studies do not report latency or energy-per-inference metrics. This is due to several factors: (i) many works prioritize algorithmic improvements or energy-management strategies rather than embedded deployment profiling; (ii) some studies rely on simulation or offline evaluation rather than real-time MCU execution; and (iii) energy and latency measurements require specialized instrumentation and standardized benchmarking procedures that are not yet consistently adopted in TinyML research. Overall, the results illustrate a clear trade-off between hardware complexity, optimization depth, and reporting completeness, reflecting the early-stage and heterogeneous nature of TinyML-driven energy management research.

### 3.4. Quality Control and Anomaly Detection in Production Lines

Quality Control (QC) and anomaly detection represent central pillars of modern industrial production systems, where uninterrupted operation, consistent product quality, and rapid detection of irregularities are essential. With the growing shift toward distributed, intelligent manufacturing, TinyML has emerged as a key technology enabling real-time decision-making at the edge. Table 8 consolidates representative studies, highlighting accuracy and F1 performance, memory footprints, inference latency, and energy consumption across diverse QC and anomaly detection tasks.

Across the quality control and anomaly detection studies (Table 8), the reported quantitative metrics reveal substantial variation in performance, hardware usage, and measurement completeness. Ref. [46] demonstrates strong performance with F1-scores of 87.6% (normal) and 96.8% (states), achieving the fastest latency at just 0.106 ms and the lowest energy consumption at 1.16 μJ per inference, although memory usage is not reported. Ref. [55] achieves high accuracy (99%) and F1 (98%), but operates on a vision-oriented platform with significantly higher latency (240/233 ms) and energy consumption (192/186 mJ), reflecting the computational cost of image-based inspection. In contrast, ref. [56] reports near-perfect accuracy (100/99.8/100) while using an extremely lightweight model (8.1 KB Flash and 0.8 KB RAM) and a modest latency of 1.16 ms, showcasing the efficiency of microcontroller-friendly architectures. Ref. [57] achieves 98% accuracy but does not provide hardware or efficiency metrics, while ref. [58] reports no quantitative results at all. Finally, ref. [59] provides detailed metrics for federated TinyML, reporting F1-scores of 97.46% (TinyFL) and 96.92% (TTFL), but with unusually high latency (16,490 ms) due to communication overheads, and an energy cost of 4.28 mJ per inference.

The missing entries across several studies likely stem from differing research priorities: many works emphasize algorithmic accuracy or federated learning design rather than embedded deployment profiling; others rely on offline or simulated evaluation rather than real-time MCU execution; and some omit latency or energy metrics due to the lack of standardized TinyML benchmarking tools. Overall, the studies illustrate a wide spectrum of design trade-offs, with some optimizing for ultra-low memory and fast inference, while others prioritize accuracy or distributed learning capabilities at the expense of computational efficiency.

### 3.5. General-Purpose TinyML Contributions for IIoT

In this section, the focus is on general-purpose TinyML contributions for IIoT that are not tightly constrained to a single industrial task but instead offer reusable architectural, algorithmic, and workflow-level solutions applicable across multiple domains. Table 9 provides a structured summary of these contributions, detailing their types, roles, relevance, and best reported results. The table illustrates how TinyML advances span deployment pipelines, distributed and federated learning, general-purpose monitoring, and compute-level optimizations, collectively enabling scalable and adaptive intelligence in industrial environments.

#### 3.5.1. Frameworks and Deployment Pipelines

Several contributions target deployment workflows and lifecycle management. Xu [36] introduces a quantization and deployment pipeline for microcontrollers, achieving 60–70% accuracy with ESP-FOMO, thereby providing a reusable workflow for MCU-based IIoT deployments. Ren [60] advances model management through online meta-learning and adaptive edge models, reporting up to a 12% accuracy improvement in dynamic environments. Complementary studies benchmark TinyML toolchains, offering comparative insights into accuracy, latency, and memory trade-offs to guide practitioners in selecting suitable frameworks [61,62]. Collectively, these contributions establish foundational enablers for scalable TinyML deployment across heterogeneous IIoT settings.

#### 3.5.2. Distributed, Federated, and Continual Learning

A second group emphasizes distributed, federated, and continual learning. Grfe [63] demonstrates distributed TinyML learning by splitting models across edge nodes, achieving an 89% latency reduction—ideal for large sensor networks. Ficco [64] integrates federated and transfer learning, reporting 86.48% accuracy while preserving privacy in decentralized IIoT systems. Llisterri-Giménez [65] extends federated learning to LoRa mesh networks, enabling low-power collaborative training in bandwidth-limited environments. Yuan [66] achieves 95.5% validation accuracy through distributed execution on dense sensor networks, while Ravaglia [67] introduces continual learning via quantized latent replay to adapt models under non-stationary operating conditions. These contributions collectively advance adaptive, decentralized TinyML intelligence for long-term industrial use.

#### 3.5.3. General-Purpose Monitoring and Sensing Applications

General-purpose monitoring and sensing applications highlight TinyML’s versatility. Zhou [33] achieves over 99% accuracy in vibration-based running-state recognition on MCUs, demonstrating portability to mobile industrial assets. Medeiros [38] reports 99.96% compression using TinyML-based vibration signal compression, reducing wireless transmission loads in structural health monitoring (SHM) systems. Katsidimas [68] achieves 98.71% accuracy in impact localization using Random Forest models on MCUs, illustrating lightweight SHM applicability. Santoro [69] develops embedded vision systems for autonomous drone landing, transferable to industrial robotics and inspection tasks. Sun [73] introduces memory-efficient anomaly detection, achieving 2–7× memory reduction and sub-0.1 s inference latency, making it suitable for real-time anomaly detection pipelines. Together, these works provide reusable design templates for monitoring and sensing across diverse industrial domains.

#### 3.5.4. Compute-Level and Architectural Optimizations

Finally, compute-level and architectural optimizations enhance TinyML efficiency. Armeniakos [37] demonstrates approximate CNN kernels, achieving 72.4% accuracy with approximate AlexNet while reducing energy consumption for vision workloads. Tabanelli [70] optimizes classical ML algorithms for ultra-low-power processors, achieving 3.7 μJ per inference, suitable for battery-powered IIoT sensors. Tabrizchi [71] introduces processing-in-sensor architectures that execute ternary MLPs (Multilayer Perceptrons) directly in sensor front-ends to reduce communication overhead. Katib [72] applies TinyML for real-time anomaly detection in IoT devices, supporting IIoT safety and device protection. These architectural innovations ensure TinyML can meet stringent resource and lifetime constraints in industrial deployments.

Overall, Table 9 shows that general-purpose TinyML contributions extend beyond specific industrial tasks to address cross-cutting concerns in deployment, adaptability, monitoring, and efficiency. Reported results (such as accuracy improvements of up to 12%, latency reductions of 89%, compression gains of 99.96%, and energy savings of 3.7 μJ per inference) demonstrate the maturity of TinyML as a scalable solution for IIoT. These contributions collectively emphasize how lightweight intelligence can be embedded across diverse industrial layers, enabling robust, efficient, and adaptive IIoT systems.

Taken together, the problem-driven classification presented in this section illustrates the breadth of TinyML applications across industrial environments and highlights how research efforts are shaped by the operational priorities of each domain. Whether targeting predictive maintenance, energy optimization, anomaly detection, or broader cross-domain sensing tasks, the reviewed studies demonstrate that TinyML is increasingly positioned as a practical enabler of real-time intelligence at the industrial edge. At the same time, the diversity of application goals underscores the need to understand the underlying technological foundations that make these solutions feasible. Building on this application-level analysis, the next section (Section 4) examines the system components (hardware platforms, software frameworks, and sensing modalities) that support TinyML deployments in IIoT settings, providing a deeper view of the architectural and implementation choices that shape performance and applicability.

## 4. System Component Taxonomy: Hardware, Frameworks, and Sensors

This section presents a structured taxonomy of the main technical components involved in TinyML–IIoT systems, focusing on hardware platforms, software frameworks, and sensing modalities.

### 4.1. Hardware Platforms and Constraints

TinyML research in IIoT uses a diverse range of embedded boards and microcontrollers, ranging from low-power BLE devices to Wi-Fi-enabled platforms with integrated accelerators. This variety reflects the need to balance energy efficiency, connectivity, and computational capability across different industrial applications and deployment scenarios. As summarized in Table 10, most studies rely on 32-bit microcontrollers from the nRF52, STM32, and ESP32 families, while others explore specialized accelerator-based platforms, heterogeneous multi-processor systems, or simulation-only environments. The table highlights the devices used, their CPU architectures, integrated accelerators, and connectivity options, providing a comprehensive view of the hardware landscape supporting TinyML deployment in industrial contexts.

Within the **nRF52 family**, studies frequently employed nRF52832 and nRF52840 microcontrollers, as well as the Arduino Nano 33 BLE Sense board. These platforms were applied to predictive maintenance, vibration-based anomaly detection, and edge learning tasks, benefiting from Bluetooth Low Energy (BLE) connectivity and floating-point unit (FPU) support when available [44,45,46,50,58,60,63,68]. Some works combined nRF52840 with RP2040 or Raspberry Pi Pico boards to compare inference behavior across heterogeneous cores, illustrating how mixed hardware can be used to benchmark TinyML performance under identical industrial scenarios.

The **STM32 family** has been used in studies evaluating TinyML model performance or supporting industrial applications that require flexible peripheral interfaces. Boards from the STM32F, STM32U, and STM32L series were used as execution environments, often without dedicated accelerators, but with FPU support and broad peripheral connectivity [34,37,38,61,62]. These platforms served as both benchmarking tools and practical deployment boards for vibration analysis, compression, and anomaly detection.

The **ESP32 and ESP8266 families** were adopted in studies emphasizing integrated wireless connectivity, including Wi-Fi and BLE. Boards such as ESP32, ESP32-S3, ESP32- CAM, and Seeed XIAO ESP32S3 (equipped with an AI accelerator) supported classification, operational-state detection, and edge-vision applications [33,36,49,66,73]. These platforms were particularly suited to distributed deployments of smart nodes that require direct network access, MQTT communication, or camera-based inspection.

For **accelerator-based and vision-oriented platforms**, specialized boards such as the OpenMV Cam H7 Plus were employed for embedded visual inspection and autonomous navigation [55,69]. GAP8 (GAPUINO) and FPGA (VCU118) platforms were explored to implement traditional machine learning algorithms on accelerator-based architectures, primarily for performance evaluation rather than full industrial deployment [70]. The Wio Terminal was also used for environmental sensing tasks, offering integrated Wi-Fi, BLE, and LCD support [56].

A subset of studies investigated **multi-board and mixed-hardware platforms**, combining devices such as the Raspberry Pi Pico W, Portenta H7, ESP32-S3, and Arduino Nano 33 BLE Sense. These setups enabled comparative evaluation across architectures (Cortex-M0+, Cortex-M4, Xtensa cores) and supported distributed learning or multi-modal sensing under realistic connectivity constraints [51,53,57,64].

In the category of **heterogeneous and multi-processor platforms**, works combined a VEGA SoC (RISC-V) with STM32 microcontrollers or integrated a Portenta H7 with LoRa modules. These systems explored continual learning and distributed inference under strict power and bandwidth limitations, demonstrating how TinyML can adapt to complex industrial environments [65,67].

Finally, several studies did not specify their hardware platforms, focusing instead on algorithmic evaluation, simulation, or benchmarking without detailed hardware reporting [47,48,52,54,71,72]. This variability in reporting limits direct comparisons across approaches but underscores the importance of transparent hardware documentation in TinyML–IIoT research.

Overall, Table 10 demonstrates that TinyML for IIoT spans a broad spectrum of hardware (from BLE-enabled microcontrollers and Wi-Fi-integrated boards to vision-oriented accelerators and heterogeneous multi-core systems). Reported results highlight trade-offs among accuracy, latency, connectivity, and energy efficiency, underscoring that hardware choice is a critical determinant of TinyML’s feasibility and scalability in industrial deployments.

### 4.2. Frameworks and Toolchains

The software frameworks adopted across TinyML–IIoT studies exhibit diversity, as summarized in Table 11. The table highlights nine distinct categories of frameworks and platforms, ranging from standardized TinyML runtimes to custom toolchains, direct on-device implementations, and simulation-based approaches. The distribution of articles across these categories illustrates both the maturity of widely adopted runtimes and the experimental nature of bespoke solutions tailored to specific industrial constraints.

The largest group of studies (13 articles) employed **custom or proprietary frameworks**, often designed to address application-specific requirements such as distributed execution, anomaly detection, continual learning, federated learning, or semantic model management [38,46,52,53,60,62,63,64,66,67,70,73]. This demonstrates TinyML’s flexibility in adapting to diverse IIoT scenarios where standardized runtimes may not suffice. The second most common framework was **TensorFlow Lite Micro (TFLM)**, appearing in 11 articles [33,34,36,44,51,55,56,57,61,62,69]. TFLM enabled the deployment of quantized neural models on memory-constrained microcontrollers and was applied to tasks such as visual inspection, gas sensing, vibration classification, and benchmarking. Its prevalence underscores its role as the de facto runtime for TinyML in IIoT. A notable subset of 10 articles relied on **direct on-device implementation** of classical machine learning models without any TinyML runtime [45,47,50,60,63,64,65,68,70,73]. These works translated decision trees, random forests, and related algorithms into lightweight C/C++ or MicroPython, achieving deterministic, low-latency execution suitable for resource-constrained industrial devices.

The **Edge Impulse platform** was used in 4 articles [33,36,49,57], providing end-to-end automation for data processing, feature extraction, model design, quantization, and code generation. Its adoption highlights the growing importance of integrated development environments that streamline TinyML workflows for vibration-based and visual pipelines. Another 4 articles employed **simulation-based approaches** [54,58,71,72], focusing on algorithmic design, optimization, or security rather than embedded inference. These studies illustrate how TinyML research often begins with off-device validation before transitioning to hardware deployment. Low-level optimization libraries such as **ARM CMSIS-NN (Cortex Microcontroller Software Interface Standard–Neural Network Library)/CMSIS-DSP (Cortex Microcontroller Software Interface Standard–Digital Signal Processing Library)** appeared in 3 articles [37,53,62], enabling efficient kernel execution on Cortex-M devices. Similarly, the **STM X-CUBE-AI toolchain** was used in 2 articles [34,62] to translate trained models into embedded inference code specific to STM32 targets. Less frequently, specialized tools such as **Imagimob Studio** (1 article) [46] were used for data preparation, while **Larq/QKeras** (1 article) [61] supported the design of highly quantized neural networks. These niche frameworks demonstrate the breadth of experimentation in TinyML software ecosystems.

Overall, Table 11 shows that software framework selection in TinyML–IIoT research is driven not only by model type but also by deployment constraints, memory budgets, communication requirements, and system-level design choices. The prevalence of custom frameworks and direct implementations reflects the need for flexibility in industrial contexts. At the same time, the widespread use of TFLM and Edge Impulse highlights the value of standardized runtimes and integrated platforms for scalable TinyML deployment.

### 4.3. Sensing Modalities

The selection of sensing modalities plays a critical role in shaping the design of TinyML systems for IIoT environments. As summarized in Table 12, the reviewed studies rely on a diverse range of sensors grouped into six main categories: motion and vibration, pressure and flow, environmental and chemical, vision and imaging, electrical and energy measurement, and acoustic and audio. Each category reflects distinct industrial requirements and constraints, and collectively, they demonstrate adaptability to heterogeneous sensing environments.

**Motion and Vibration Sensors** represent the most widely adopted modality, appearing in nine studies [33,38,44,47,49,50,58,68,73]. These include accelerometers, gyroscopes, IMUs, and piezoelectric sensors, which are central to predictive maintenance, mechanical condition monitoring, and human-motion assessment. Applications range from rail-vehicle state recognition and bearing-fault detection to multimodal pump diagnostics and wearable lifting-risk evaluation. In some cases, piezoelectric transducers simultaneously provide vibration sensing and energy harvesting, underscoring their dual utility in battery-less TinyML nodes.

**Pressure and Flow Sensors** are less common but highly relevant for industrial process monitoring. Martinez-Rau [46] employed hydraulic and differential pressure sensors, along with motor-speed measurements, to characterize periodic machine duty cycles. This modality enables effective fault identification without requiring high-bandwidth vibration data, offering a lightweight alternative for process diagnostics.

**Environmental and Chemical Sensors** appear in three studies [50,56,66], supporting contextual awareness and hazardous condition detection. These include gas sensors, temperature, and humidity sensors. Applications range from collaborative environmental monitoring across distributed TinyML nodes to pump diagnostics using thermal indicators, and VOC classification using MOS-based gas sensor arrays. Their low-bandwidth nature makes them particularly suitable for microcontroller-class devices.

**Vision and Imaging Sensors** are employed in three studies [55,57,69], enabling spatial and visual understanding in industrial contexts. RGB cameras support defect detection in manufacturing and drone-landing guidance, while infrared cameras provide thermal imaging for fault detection in photovoltaic modules. These modalities introduce substantial data volume, requiring efficient preprocessing and optimized CNN architectures for deployment on constrained hardware.

**Electrical and Energy Measurement Sensors** are central to energy profiling and battery modeling, appearing in two studies [51,53]. Voltage and current sensors, along with fuel-gauge ICs and ADC-based measurements, enable real-time state-of-charge estimation and detailed energy-consumption profiling. These signals support TinyML models that infer device power behavior directly from electrical observations, advancing energy-aware edge intelligence.

**Acoustic and Audio Sensors** are reported in two studies [48,65], spanning low-power audio command recognition and high-frequency structural analysis. Microphones enable federated learning in LoRa-based networks, while acoustic emission sensors capture ultrasonic waveforms associated with the propagation of material damage. Despite differences in bandwidth and computational requirements, both modalities provide valuable signals for TinyML applications.

Overall, Table 12 demonstrates that motion and vibration sensors dominate mechanical monitoring tasks, while pressure, environmental, vision, electrical, and acoustic sensors address specialized but equally important IIoT use cases. Sensor choice directly shapes model design, feature-extraction strategies, and the feasibility of performing efficient on-device inference under strict energy and latency constraints. The diversity of modalities underscores TinyML’s flexibility in adapting to diverse industrial environments, enabling robust, context-aware intelligence across IIoT systems.

To summarize, this section highlights how TinyML deployments in IIoT environments are shaped by the interplay between hardware capabilities, software frameworks, and sensing modalities. The reviewed studies demonstrate that system-level design choices (ranging from microcontroller architectures and memory constraints to framework support and sensor characteristics) directly influence the feasibility, efficiency, and robustness of TinyML solutions. These components form the technological foundation upon which application-specific intelligence is built, and they determine the practical limits of model complexity, latency, and energy consumption at the edge. Having established this system-level landscape, the next step is to examine how researchers employ datasets, model architectures, and optimization strategies to operationalize TinyML within these constraints. This methodological perspective is essential for understanding how TinyML solutions are engineered to meet industrial requirements in real-world IIoT scenarios.

## 5. Methodologies: Datasets, Model Architectures, and Optimization Strategies

Building on the system-level foundations outlined in Section 4, which examined the hardware platforms, software frameworks, and sensing modalities enabling TinyML deployment in IIoT environments, this section shifts the focus toward the methodological layer of these systems. While system components determine what is technically feasible at the edge, the effectiveness of TinyML solutions ultimately depends on the datasets, model architectures, and optimization strategies used to meet stringent industrial constraints. This section therefore synthesizes the methodological choices observed across the reviewed studies, highlighting how data characteristics, model families, and resource-aware optimization techniques shape performance, scalability, and applicability in real-world IIoT scenarios.

### 5.1. Dataset Characteristics and Challenges

The reviewed TinyML–IIoT literature reveals substantial diversity in the datasets used to train and evaluate embedded machine learning models. To enhance clarity and interpretability, the datasets are organized into two complementary categories: (1) operational and real-world sensor datasets, which represent measurements collected from physical systems, industrial environments, or controlled laboratory experiments; and (2) public, benchmark, and synthetic datasets, which encompass widely used open datasets, standardized benchmarks, and simulated datasets designed for experimentation. Table 13 and Table 14 summarize these datasets, highlighting their sources, modalities, and sizes.

#### 5.1.1. Operational and Real-World Sensor Datasets

Table 13 lists 18 studies that rely on datasets collected directly from industrial processes, mechanical systems, environmental sensing networks, or human participants. These datasets capture natural variability, sensor noise, and operational fluctuations, thereby closely mirroring deployment conditions in IIoT environments. A significant subset originates from **motion and vibration sensing**, which underpins predictive maintenance and structural health monitoring. Examples include piezoelectric vibration signals for self-powered sensing [44], IMU-based pump monitoring [50], triaxial vibration measurements for motor diagnostics [49], and impact-event waveforms totaling 771 instances with 5000 samples each [68]. Rail-vehicle running-state recognition [33] and bearing fault detection [47] further demonstrate the centrality of vibration datasets in TinyML research.

Other datasets capture **operational industrial signals**, such as duty-cycle sequences from conveyor systems [45], hydraulic pressure and motor-speed signals comprising 170,000 samples and 603 cycles [46], and time-series measurements collected from real IIoT installations for anomaly detection [73]. These datasets enable models to learn system-level patterns across extended operating cycles. Several studies incorporate **vision-based datasets** sourced from industrial or environmental contexts. Examples include infrared thermography images for PV-panel inspection (2000 images) [57], RGB images for manufacturing quality assessment [55], drone-landing imagery exceeding 26,000 labeled samples [69], and industrial object images captured under diverse lighting conditions [36]. These datasets highlight the growing role of embedded vision in TinyML–IIoT.

Additional datasets include **energy and electrical measurements**, such as profiling traces for embedded power consumption [53] and battery voltage, current, and temperature readings from Li-ion cycling experiments [51]. **Acoustic datasets** include ultrasonic AE waveforms for damage assessment in turbine blades [48], while **human-centric datasets** include IMU data for ergonomic movement evaluation [58]. Environmental IoT sensor readings from ESP32 networks [66] and bridge vibration/strain signals from Queen’s Park Bridge [38] further expand the scope. Collectively, these datasets embody realistic sensor conditions and operational challenges, serving as essential testbeds for evaluating TinyML models under deployment-level constraints. Although several studies utilize datasets collected from real industrial systems and operational environments, many TinyML–IIoT studies are still validated primarily through laboratory experiments, prototype platforms, or controlled testbeds. In several cases, public benchmark datasets or custom experimental datasets are used instead of continuous industrial data streams from large-scale deployments. This observation reflects the current maturity level of TinyML applications in industrial IoT, where real-world deployments remain relatively limited compared to laboratory-based evaluations.

#### 5.1.2. Public, Benchmark, and Synthetic Datasets

Table 14 summarizes 15 studies that rely on publicly available benchmarks, standardized evaluation suites, or synthetic datasets. These datasets complement real-world collections by providing reproducible experimental conditions, controlled variability, and standardized baselines for comparing lightweight learning models.

**Image datasets** are widely used, including CIFAR-10 [37,67], MNIST and Fashion- MNIST [71], CBCL Face [71], and mixed benchmarks such as CIFAR-100 and Visual Wake Words (VWW) [62]. These datasets, ranging from 60,000 to 70,000 samples, support embedded vision experiments and quantized CNN evaluations. **Audio and speech datasets** include Speech Commands, keyword-spotting corpora, and machine-sound anomaly datasets (ToyADMOS), appearing in [60,62,65,67,70]. Dataset sizes vary from 480 samples (GitHub KWS) to over 105,000 samples (Speech Commands), enabling systematic evaluation of TinyML models for low-power speech recognition and sound classification. **Time-series datasets** are also prominent. The UCR Archive supports generic signal classification [63], while NASA C-MAPSS provides degradation and Remaining Useful Life (RUL) data [34]. Bearing vibration datasets (Afshar, 10,000-sample windows) [47], ECG datasets (MIT-BIH, PTB) [64], and mobility datasets (Car Trips, DeepEST with 45,500 samples) [64] extend TinyML applications to health monitoring and operational diagnostics. The ToN-IoT benchmark dataset (23,500 instances) [72] supports anomaly detection in networked IoT systems, while PAMAP2, SHL (750 h), and CWRU datasets [61] provide activity and vibration data for embedded classification tasks.

Finally, **synthetic datasets** are employed when real industrial data are unavailable. Examples include process simulations for underground mining networks [54] and task-energy simulations for edge-scheduling optimization [52]. These datasets facilitate controlled experimentation, allowing researchers to analyze specific system behaviors and evaluate optimization strategies under repeatable conditions.

Overall, Table 13 and Table 14 demonstrate that TinyML–IIoT research leverages both real-world sensor datasets and standardized benchmarks. Real-world datasets capture operational variability and deployment challenges, while public and synthetic datasets provide reproducibility and controlled baselines. Together, they enable comprehensive evaluation of TinyML models, balancing practical deployment validation with systematic benchmarking and generalization assessment.

### 5.2. Model Architectures

**Figure 5:**
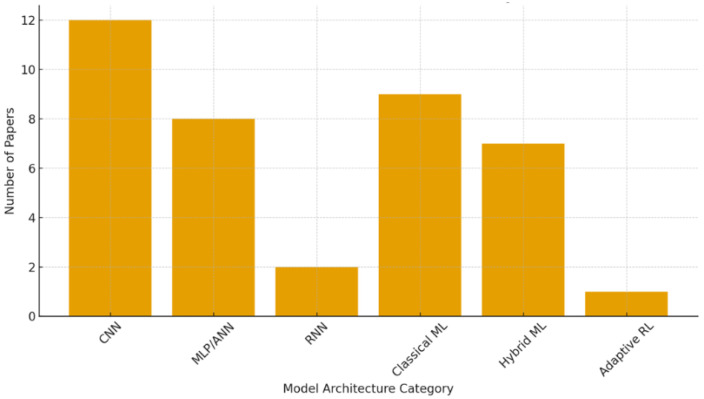
Distribution of model architectures used in reviewed TinyML–IIoT studies.

The distribution of model architectures across the reviewed TinyML–IIoT studies shows a clear hierarchy of model types favored for deployment on resource-constrained embedded platforms. As summarized in Table 15 and visualized in Figure 5, CNNs constitute the most prevalent architecture, appearing in 12 studies. This is followed by classical machine-learning models and Multilayer Perceptron (MLP)/Artificial Neural Network (ANN) architectures. In contrast, Recurrent Neural Network (RNN)/Long Short-Term Memory (LSTM) networks, hybrid architectures, and reinforcement-learning models appear less frequently. This distribution reflects the practical constraints of IIoT environments, where model size, latency, and energy footprint often take precedence over representational depth.

CNNs are the dominant architecture category due to their strong performance in extracting local spatial or temporal patterns while remaining relatively compact after quantization. They are widely used in tasks such as image-based inspection, thermal analysis, and vibration classification. For instance, lightweight CNN variants are deployed in embedded vision systems for quality inspection [55], IR thermal analysis [57], and drone landing guidance [69]. Optimized 1D-CNNs are also employed in vibration-based fault diagnosis on ESP32 devices [47,49], while other works evaluate CNN efficiency under model-compression schemes [34] or across industrial TinyML toolchains [61]. Collectively, these studies demonstrate that CNNs offer a favorable balance between accuracy and computational feasibility for TinyML deployments.

MLP-based architectures appear in 9 studies and are preferred for low-dimensional sensor signals or tabular industrial data. Their fully connected structure is computationally predictable and manageable to quantize, making them suitable for distributed TinyML computation [66], acceleration-based state classification [33], gas sensing [56], and energy profiling [53]. Other studies embed MLPs directly into sensor front-ends [71] or use them within federated and meta-learning frameworks [60,64,65]. These architectures highlight the role of shallow neural networks as efficient alternatives to CNNs when spatial feature extraction is unnecessary.

RNN-based architectures are the least represented, appearing in only two studies. Their primary use is in modeling sequential dependencies in electrical signals—for example, LSTM networks for battery State-of-Charge estimation [51]. Despite their modeling strengths, the memory and computational demands of recurrent layers make them less common in TinyML deployments than convolutional or feedforward models.

Classical ML remains highly competitive in TinyML due to its low computational cost and interpretability. Nine studies employ models such as Random Forests, SVMs, KNN, Gradient Boosting, or Isolation Forests. These architectures are used for anomaly detection [44,50,73], industrial duty-cycle classification [45,46], structural monitoring [38,68], or distributed learning pipelines [63]. Classical ML is particularly attractive when features can be engineered manually, enabling microsecond-scale inference and extremely low energy consumption.

Hybrid architectures appear in seven studies and integrate deep learning feature extractors with classical ML decision layers. Examples include CNN–SVM pipelines for acoustic-emission structural monitoring [48], ternary MLPs embedded directly in sensor hardware [71], and meta-learning or online-learning extensions built on top of compact neural backbones [60]. Other studies combine optimization algorithms with ML components for decision support [54]. These hybrid strategies illustrate how TinyML can be tailored to specialized industrial constraints by mixing architectural paradigms.

RL-based architectures appear rarely, with only one study adopting an adaptive RL agent to optimize edge-computing latency and energy consumption [52]. While RL is promising for dynamic resource management, it is not yet common in embedded TinyML due to the computational overhead of policy updates.

Overall, the architectural landscape shows a clear trend: CNNs are preferred for high-dimensional sensing tasks, while classical ML models and compact MLPs remain the most practical choice for heavily constrained microcontrollers. Recurrent and hybrid architectures appear only in specialized scenarios that require temporal modeling or multi-stage processing.

### 5.3. Optimization Technique

Optimization techniques are essential for enabling the deployment of TinyML models under the strict memory, latency, and energy constraints of IIoT devices. As summarized in Table 16, the reviewed studies identify six main optimization categories: Post-Training Quantization, Pruning/Weight Reduction, Approximate Computing, Meta-Learning/Online Optimization, Reinforcement Learning Optimization, and Metaheuristic Optimization. Each technique contributes uniquely to reducing model size, lowering computational costs, or improving system-level efficiency.

**Post-Training Quantization** is the most frequently adopted optimization approach, appearing in five studies [34,47,49,55,56]. By reducing numerical precision (e.g., INT8 quantization), these works demonstrate significant memory savings and latency reductions with minimal accuracy loss. Applications span vibration-based fault diagnosis on ESP32 platforms, embedded vision inspection using quantized MobileNetV2 and SqueezeNet, and CNN-based remaining useful life estimation. Quantization thus emerges as a foundational optimization technique for TinyML deployment in IIoT.

**Pruning and Weight Reduction** is reported in three studies [34,49,55], where redundant parameters are removed, or matrix factorization is applied to streamline models. These approaches reduce computational overhead and memory footprint while maintaining accuracy. For example, pruning combined with weight clustering reduced CNN parameters by up to 58% in [34], while embedded vision tasks in [55] benefited from pruning-driven efficiency gains. In vibration anomaly detection, pruning improved inference latency on ESP32S3 hardware [49].

**Approximate Computing** appears in [37], where exact convolution kernels are replaced with approximate multiply–accumulate (MAC) operations. This approach achieved up to 30% inference latency reduction while maintaining accuracy within 1%. Approximate computing demonstrates that algorithmic approximation can complement or substitute traditional quantization–pruning pipelines, offering a novel paradigm for energy-efficient TinyML.

**Meta-Learning and Online Optimization** is represented by [60], which introduces TinyOL, TinyReptile, and TinyMetaFed. These methods focus on adaptability in dynamic IIoT environments, optimizing energy usage, communication overhead, and training iterations rather than model size. Reported gains include 50% energy savings and 60% communication reduction, highlighting the importance of adaptive learning strategies for resource-constrained deployments.

**Reinforcement Learning Optimization** is employed in [52], where an RL-based scheduler dynamically distributes tasks at the edge. This adaptive scheduling mechanism improved energy efficiency by 38% and reduced system latency compared to static scheduling. RL optimization thus extends TinyML efficiency beyond the model level to system-level orchestration.

**Metaheuristic Optimization** is reported in [54], which applied the Aquila Optimization Algorithm (AOA) to improve compression ratios and routing efficiency in IIoT networks. The approach achieved a 29% reduction in energy consumption and an 18% increase in compression gain, demonstrating the value of global optimization strategies in communication-intensive industrial settings.

However, selecting an appropriate optimization strategy involves important trade-offs. Techniques such as quantization and pruning reduce memory footprint and inference latency, but aggressive compression may lead to accuracy degradation or reduced robustness. Similarly, methods that improve model accuracy or adaptability may increase computational complexity and energy consumption. Consequently, TinyML optimization in IIoT systems requires careful balancing of accuracy, latency, and energy efficiency to meet the operational constraints of resource-constrained microcontrollers. Overall, Table 16 shows that TinyML optimization techniques span multiple levels—from precision reduction and parameter pruning at the model level to adaptive scheduling and heuristic optimization at the system level. Quantization and pruning dominate due to their direct impact on memory and latency, while approximate computing, meta-learning, reinforcement learning, and metaheuristics expand the optimization landscape to address adaptability, energy efficiency, and communication overhead. Despite methodological differences, all approaches converge toward the shared goal of enabling efficient, accurate, and real-time intelligence on resource-constrained IIoT microcontrollers.

To provide a concise synthesis of the methodological patterns observed across the reviewed studies, we include Table 17, which summarizes the relationship between (i) the dominant data types used in TinyML–IIoT systems, (ii) the model families most commonly applied to these data, and (iii) the optimization techniques typically employed to enable deployment on resource-constrained microcontrollers. This table does not replace Table 13, Table 14, Table 15 and Table 16; instead, it complements them by offering a high-level mapping derived from their contents. Table 13 and Table 14 describe dataset characteristics, Table 15 summarizes model architectures, and Table 16 outlines optimization strategies. Bringing all of this information into a single detailed table would introduce redundancy and reduce readability. Therefore, the following summary table provides a compact, integrative view that captures the essential relationships without duplicating the detailed information already presented in the earlier tables.

Section 5 demonstrates how TinyML methodologies span datasets, model architectures, and optimization strategies across IIoT contexts. However, these methodological insights also reveal important limitations that currently restrict large-scale industrial deployment. These limitations are not isolated to specific techniques but emerge across sensing, hardware, software, and deployment workflows. Therefore, the next section synthesizes the key challenges that persist across the reviewed studies. The objective is to highlight the technical and operational gaps that remain unresolved. It also outlines the issues that must be addressed to enable robust, scalable, and energy-efficient industrial deployments.

## 6. Challenges and Future Directions

Despite the growing maturity of TinyML in IIoT environments, several challenges continue to limit its scalability, reliability, and long-term applicability. Section 6.1 discusses these challenges, while Section 6.2 outlines promising solutions for these challenges.

### 6.1. Challenges

These challenges arise from hardware constraints, energy limitations, dataset issues, and the absence of standardized evaluation practices. The following paragraphs elaborate on these issues in detail.

**Memory and Compute Limits:** As shown in Table 10, microcontrollers such as ESP32 and nRF52 typically provide less than 1 MB of RAM and modest clock speeds. This restricts the depth and complexity of deployable models. Studies on vibration monitoring and real-time diagnostics confirm that only compact CNNs or classical ML models can be executed reliably (Section 3). The results in Section 5.3 show that quantization and pruning are widely used to fit models into constrained devices [23,24]. However, these techniques often reduce accuracy, especially in tasks requiring fine-grained anomaly detection [25,27]. This highlights the persistent trade-off between efficiency and reliability in TinyML-enabled IIoT deployments [28].

**Energy Constraints:** Battery-powered IIoT systems face strict energy budgets, which directly limit the feasibility of continuous TinyML inference [12]. Chen et al. [44] highlight that prolonged inference significantly reduces battery life, especially in vibration monitoring tasks where high-frequency sampling is required. The systematic review confirms that most deployments adopt periodic inference or compressed data transmission to conserve energy [19]. While this strategy reduces communication overhead and extends device lifetime, it compromises responsiveness in time-critical applications such as predictive maintenance and anomaly detection [25]. Earlier reviews [23,24] also emphasize that energy efficiency remains one of the most persistent barriers to scaling TinyML in IIoT.

**Harsh Industrial Conditions:** TinyML deployments in industrial environments must also address physical and environmental challenges that are rarely captured in laboratory experiments. Industrial systems often operate under harsh conditions, including high temperatures, mechanical vibration, electromagnetic interference, and significant sensor noise. These factors can affect both sensor reliability and the stability of embedded inference systems, introducing measurement variability that may degrade model accuracy and robustness [33,38,50,73]. Consequently, improving model resilience to noisy data, enhancing sensor calibration, and designing fault-tolerant pipelines remain important research directions in IIoT environments.

**Weak Hardware Accelerator Support:** Most general-purpose processing devices lack dedicated matrix-operations accelerators, which makes the execution of CNNs and hybrid models inefficient (Table 10). As a result, inference times are longer, and energy consumption per operation is higher, limiting the feasibility of real-time deployments [12]. Only a few platforms integrate specialized accelerators, such as ARM Ethos-U, but these remain underutilized in industrial prototypes due to cost and limited availability. The systematic review confirms that most deployments rely on software-optimized runtimes such as TensorFlow Lite Micro and CMSIS-NN, which provide portability but only modest speedup [23,24]. In predictive maintenance and vibration analysis tasks, this limitation forces researchers to adopt compact CNNs or classical ML models that can run on general-purpose devices, but at the expense of accuracy and scalability [19]. Future research must therefore explore co-design approaches that align hardware capabilities with algorithmic requirements, including lightweight accelerators for convolutional operations and attention mechanisms.

**Limited On-Device Learning:** Continual learning and adaptive inference remain largely unsupported on microcontrollers, limiting the ability of TinyML systems to adjust to evolving industrial conditions. Although Ren [60] demonstrates lightweight adaptation strategies, these approaches are still experimental and introduce non-trivial energy and memory overheads that exceed the capabilities of most IIoT devices. The systematic review shows that current TinyML deployments rely predominantly on offline retraining and periodic cloud synchronization [23,24], reducing adaptability in environments where sensor characteristics and machine states change frequently. Applications such as vibration-based predictive maintenance [19] require models to accommodate new fault signatures, yet the absence of efficient on-device learning causes these systems to become static over time. Similarly, anomaly detection in IIoT [25] highlights the need for adaptive inference, but most implementations remain cloud-centric. Broader manufacturing reviews [27,28] also prioritize interpretability over adaptability, leaving continual learning on embedded devices largely unexplored. Without practical on-device learning mechanisms, TinyML-enabled IIoT systems risk reduced robustness, slower responsiveness to evolving operational contexts, and limited scalability across diverse industrial domains.

**Accuracy–Latency–Size Trade-offs:** Balancing accuracy, latency, and memory footprint remains a central challenge in TinyML-enabled IIoT systems. Compact models often reduce predictive accuracy to meet strict memory and timing constraints, especially on microcontrollers with less than 1 MB of RAM [12]. This trade-off is particularly critical in high-frequency sensing tasks such as structural monitoring and anomaly detection, where reduced accuracy can compromise safety [25]. Although quantization and pruning improve efficiency, several reviews report that these techniques frequently degrade accuracy [23,24]. The systematic review further shows that while lightweight CNNs and classical ML models are feasible for vibration-based predictive maintenance [19], they often struggle to capture complex industrial patterns, limiting robustness in safety-critical contexts. Broader manufacturing surveys [27,28] also highlight that lightweight models face persistent tension between inference speed and predictive performance. These challenges underscore the need for integrated co-design approaches that jointly optimize algorithms, hardware, and sensing modalities to achieve reliable accuracy under stringent resource constraints.

**Lack of Standardized Metrics:** Performance reporting remains inconsistent across studies, which makes it challenging to compare TinyML deployments in IIoT environments. Hasanpour [62] emphasizes the absence of unified benchmarks for TinyML, noting that current evaluation practices are fragmented and often incomplete. Some studies report latency, energy consumption, or memory footprint, but the metrics vary widely across publications and lack common definitions [23,24]. Even when benchmarks are used, they often fail to capture industrial constraints such as sensor noise, workload variability, and long-term reliability [25]. For example, predictive maintenance studies based on vibration sensing [19] report accuracy and inference speed but rarely include energy-per-inference or device lifetime performance metrics. Reviews of ML in manufacturing [27,28] also highlight inconsistencies in reporting, with some focusing on interpretability while ignoring resource efficiency. This inconsistency hampers reproducibility and comparability, making it difficult to evaluate progress across different deployments and to identify best practices for industrial adoption. Future work must therefore establish standardized evaluation scenarios and unified benchmarks that reflect realistic industrial conditions, including noisy data streams, heterogeneous hardware, and long-term operational monitoring.

**Dataset Limitations:** Most studies rely on small, domain-specific datasets, which restrict the generalizability of TinyML models to broader industrial contexts. While benchmarks aid reproducibility, they often lack realism and diversity, failing to capture the variability of real-world industrial environments [23,24]. This limitation is particularly evident in predictive maintenance and vibration analysis tasks, where datasets are narrow in scope and do not reflect long-term operational conditions [19]. The systematic review results show that vibration datasets dominate current research, while other modalities such as vision, acoustic, and environmental sensing remain underexplored [25]. As a result, TinyML models risk overfitting and poor performance when deployed in heterogeneous IIoT scenarios. Reviews of ML in manufacturing [27,28] also highlight that dataset scarcity and lack of diversity hinder interpretability and scalability, reinforcing the need for richer multimodal datasets. Without larger, more representative datasets, TinyML-enabled IIoT systems will continue to face challenges achieving robust, transferable, and reliable performance across diverse industrial domains. In addition, industrial datasets often exhibit non-independent and non-identically distributed (non-IID) characteristics due to variations in machines, operating conditions, and sensor configurations across different industrial environments. As a result, models trained on data from a specific system may not generalize well to other deployments. Combined with limited failure examples and class imbalance, these non-IID properties further complicate the development of robust TinyML models for real-world IIoT applications.

**Safety and Security of TinyML in IIoT Systems:** Ensuring the safety and security of TinyML-enabled IIoT systems remains a critical challenge, particularly because these models operate on resource-constrained devices deployed in physically exposed and operationally sensitive industrial environments. Unlike cloud-based AI, TinyML models run locally on microcontrollers, making them more vulnerable to adversarial manipulation, model extraction, firmware tampering, and physical attacks. The limited computational and memory resources of processing devices further restrict the use of conventional security mechanisms such as encryption, secure boot, or runtime anomaly detection [37,67]. Safety risks also arise when compromised or malfunctioning on-device models generate incorrect predictions that influence real-time industrial decisions, potentially affecting equipment reliability or worker safety. Recent work on federated and on-device learning highlights additional challenges related to secure model updates, privacy preservation, and resilience against poisoning attacks in distributed industrial environments [52,74]. Addressing these concerns requires lightweight security frameworks, robust model-integrity verification, secure update mechanisms, and safety-aware model design tailored to the constraints of TinyML hardware. As TinyML adoption grows, integrating security-by-design principles will be essential to ensure trustworthy and resilient IIoT deployments.

**Overall Insight:** The SLR highlights that TinyML in IIoT remains promising but incomplete. Hardware bottlenecks, energy constraints, weak adaptability, dataset scarcity, and inconsistent evaluation continue to limit its scalability and reliability. Addressing these challenges requires co-design approaches that integrate hardware, software, and algorithmic innovations. It also demands standardized benchmarks and richer datasets to ensure reproducibility and industrial relevance.

### 6.2. Future Directions

Addressing the identified challenges opens several promising research directions that can significantly advance TinyML’s role in industrial systems. These directions build directly on the limitations of current deployments, including hardware constraints, limited datasets, limited adaptability, and inconsistent evaluation practices. The following areas highlight opportunities for improvement and innovation.

**Advanced Model Optimization Techniques:** Memory and compute limits remain significant barriers to TinyML deployment on microcontrollers such as the ESP32 and STM32. Future work should explore quantization-aware training, structured pruning, and approximate computation to reduce model size while maintaining accuracy [37]. The systematic review shows that current approaches rely heavily on post-training quantization and pruning, which often degrade accuracy. Earlier reviews [23,24] emphasized compression and co-design strategies but lacked industrial validation. Integrating optimization into the training process and aligning it with hardware-aware scheduling can better balance accuracy, latency, and memory footprint, especially for vibration monitoring and anomaly detection tasks.

**Meta-Learning and On-Device Adaptation:** Limited support for continual learning restricts adaptability in dynamic industrial environments. Meta-learning and lightweight on-device adaptation methods can enable models to update locally with minimal communication overhead [60]. This would reduce reliance on cloud retraining and improve robustness under non-stationary conditions such as equipment wear or environmental changes. The review results confirm that most current deployments depend on offline retraining, highlighting the need for efficient adaptation strategies. Recent studies on predictive maintenance [19] show the potential of vibration-based adaptation, but broader IIoT domains remain unexplored. Future research should also explore federated learning approaches tailored for microcontrollers, allowing distributed updates across multiple devices without compromising energy efficiency.

**Development of Realistic Industrial Datasets:** Dataset limitations remain a critical issue, with most studies relying on small, domain-specific datasets. Future research should focus on building larger and more realistic datasets for vibration analysis, multi-sensor fusion, and long-term operational monitoring. Such datasets would improve generalizability and reduce overfitting, ensuring that TinyML models perform reliably in diverse industrial contexts. The systematic review found that vibration sensing dominates current research [19], while modalities such as vision and acoustic sensing are underexplored. Expanding dataset diversity will strengthen the applicability of TinyML across multiple IIoT domains. Collaborative dataset initiatives, where industries share anonymized sensor data, could accelerate progress and provide benchmarks that reflect real deployment conditions.

**Standardized Evaluation Scenarios and Benchmarks:** The lack of standardized performance metrics hampers reproducibility and comparability across studies. Emerging frameworks such as EdgeMark [62] provide a foundation for unified evaluation of latency, energy consumption, and robustness. Future work should extend these benchmarks to cover realistic industrial scenarios, including noisy environments, variable workloads, and long-term reliability testing. Standardization will ensure that reported results are consistent and meaningful, accelerating progress in TinyML research and deployment. Earlier reviews [25,26,27] highlighted inconsistency in reporting ML performance in IIoT, underscoring the need for unified TinyML-specific benchmarks.

**Creation of Domain-Specific IIoT Scenarios:** Many current studies emphasize algorithmic improvements without demonstrating integration into actual industrial workflows. Future research should prioritize domain-specific IIoT scenarios, such as predictive maintenance pipelines, energy-optimization systems, and quality-inspection processes. Application-grounded validation will bridge the gap between experimental prototypes and real-world deployments. The systematic review highlights that most TinyML studies remain proof-of-concept, underscoring the need for more industrially relevant case studies. Reviews on ML in IIoT [27,28] categorized techniques broadly but did not address TinyML edge deployment. Developing end-to-end prototypes that integrate sensing, inference, and decision-making in real factory environments will provide more substantial evidence of TinyML’s industrial value.

**Enhanced Hardware Support:** Weak accelerator support continues to limit inference speed and energy efficiency on general-purpose MCUs. Future hardware platforms should integrate native accelerators and low-power AI engines to expand the range of feasible TinyML workloads. Such hardware would reduce the gap between experimental models and deployable industrial solutions. The review results show that current deployments rely on software-optimized runtimes such as TensorFlow Lite Micro and CMSIS-NN, which offer portability but limited speedups. Enhanced hardware support will enable more complex models to run efficiently in latency-sensitive IIoT applications. Co-design approaches that align hardware capabilities with algorithmic requirements can maximize efficiency. For example, specialized accelerators for convolutional operations or attention mechanisms could unlock new industrial applications such as vision-based quality inspection or multimodal sensor fusion.

**Overall Outlook:** Together, these future directions address the core challenges of TinyML in IIoT: hardware bottlenecks, energy constraints, dataset scarcity, weak adaptability, and inconsistent evaluation. Advances in optimization, adaptation, dataset development, benchmarking, domain-specific validation, and hardware design will enable TinyML to evolve from experimental prototypes into scalable, reliable, and energy-efficient industrial solutions. By bridging the gap between research and deployment, TinyML can become a cornerstone of Industry 4.0, supporting predictive maintenance, smart energy management, and autonomous industrial operations.

The challenges and future directions outlined above provide essential context for interpreting the findings of this review. To consolidate these insights and directly address the objectives defined in Section 1.4, the following section synthesizes the results in relation to the five research questions. This structured synthesis links the evidence gathered across applications, system components, methodologies, and limitations, offering a clear and comprehensive response to the core aims of this SLR.

## 7. Answers to Formulated Research Questions and Limitations of the SLR

Building on the methodological insights presented in Section 5 and the challenges and future directions discussed in Section 6, this section synthesizes the overall findings of the SLR by answering the five research questions defined in Section 1.4. These answers integrate evidence from application domains, system components, datasets, model architectures, and optimization strategies, providing a unified perspective on the current state and future potential of TinyML in IIoT environments. Answers in Section 7.1 are based on the synthesis of selected studies and supported by summary tables. Section 7.2 then outlines methodological limitations to ensure transparency.

### 7.1. Answers to Formulated Research Questions

*RQ1: In which industrial domains and operational tasks has TinyML been applied within IIoT systems?*

Answer: The reviewed studies show that TinyML has been applied across multiple IIoT domains, primarily for operational tasks that require low-latency, energy-efficient on-device intelligence. As summarized in Table 4, the most common application areas include smart manufacturing and predictive maintenance, industrial equipment and condition monitoring, smart energy management, and quality control with anomaly detection in production lines. Predictive maintenance and condition monitoring applications dominate the literature, where TinyML models process vibration and sensor-based time-series data to enable early fault detection and localized diagnostics (Table 5 and Table 6). In smart energy management, TinyML is mainly used for energy profiling and efficiency optimization under strict resource constraints (Table 7). Quality control and anomaly detection applications leverage embedded vision and sensor data to perform real-time inspection directly on industrial devices (Table 8). Several studies address general TinyML applications in IIoT, focusing on deployment workflows and transferable solutions applicable across multiple industrial scenarios (Table 9). Overall, these findings highlight TinyML’s suitability for IIoT environments where local decision-making is essential and cloud-based analytics are constrained by latency or energy limitations.

*RQ2: What system components (hardware platforms, software frameworks, and sensor modalities) are most commonly used for deploying TinyML in IIoT?*

Answer: The reviewed studies show that TinyML deployment in IIoT environments primarily relies on low-power microcontroller platforms, reflecting the stringent resource and energy constraints of industrial edge devices. As summarized in Table 10, microcontroller families such as nRF52, STM32, and ESP32/ESP8266 are the most frequently used, owing to their balance between computational capability, energy efficiency, and peripheral support. Accelerator-based and vision-oriented platforms (e.g., GAP8, FPGA, OpenMV Cam) are less common and are mainly used in application-specific scenarios involving image or vision-based processing. Multi-board and heterogeneous platforms (e.g., Portenta H7, Raspberry Pi Pico W, VEGA SoC) are also explored to evaluate distributed inference and continual learning under realistic connectivity constraints. From a software perspective, the literature shows a clear concentration around a small set of widely adopted frameworks. As reported in Table 11, TensorFlow Lite Micro (TFLM), direct on-device implementations, and custom proprietary frameworks are the most commonly used solutions, supported by their flexibility and suitability for resource-constrained hardware. Platforms such as Edge Impulse and low-level libraries like ARM CMSIS-NN/CMSIS-DSP are also employed, particularly where streamlined deployment workflows and optimized inference kernels are required. Other toolchains such as STM X-CUBE-AI, Imagimob Studio, and Larq/QKeras appear in niche cases, reflecting experimentation with specialized workflows. Sensor integration plays a central role in hardware selection. As summarized in Table 12, motion and vibration sensors (accelerometers, gyroscopes, IMUs, piezoelectric devices) dominate predictive maintenance and condition monitoring tasks. Other modalities include pressure and flow sensors for process monitoring, environmental and chemical sensors for hazardous detection, vision sensors for defect inspection, electrical sensors for energy profiling, and acoustic sensors for audio-based anomaly detection. The diversity of sensing modalities directly shapes model design, feature extraction strategies, and the feasibility of efficient on-device inference.

*RQ3: What methodologies (datasets, model architectures, and optimization strategies) in TinyML–IIoT techniques are employed to adapt ML models for resource-constrained IIoT devices?*

Answer: The reviewed studies reveal substantial diversity in datasets, architectures, and optimization strategies used to adapt ML models for IIoT devices. Datasets are organized into two categories: operational and real-world sensor datasets (Table 13) and public, benchmark, and synthetic datasets (Table 14). Model architectures show a clear hierarchy (Table 15). CNNs dominate (12 studies) for vibration analysis, image inspection, and thermal imaging due to their compactness after quantization. Classical ML models (9 studies), such as Random Forests, SVMs, and KNN, remain attractive for anomaly detection and condition monitoring due to their low computational cost. MLPs/ANNs (9 studies) are used for low-dimensional sensor signals and distributed learning pipelines. Hybrid architectures (7 studies) combine CNNs or MLPs with classical ML layers for specialized tasks, while RNN/LSTM models (2 studies) are mainly used for sequential battery state estimation. Adaptive RL is rare, with only one study applying it for edge scheduling optimization. Optimization strategies (Table 16) are critical for deployment under strict resource constraints. Post-training quantization is the most frequent technique, reducing memory and latency with minimal accuracy loss. Pruning and weight reduction streamline CNNs and vision models by removing redundant parameters. Approximate computing achieves latency reductions by replacing exact MAC operations with approximate kernels. Meta-learning and online optimization improve adaptability in dynamic IIoT environments, while reinforcement learning optimization enables adaptive scheduling for energy-efficient edge computing. Metaheuristic optimization applies global search strategies to improve routing and compression efficiency in IIoT networks.

*RQ4: How is the performance of TinyML models in IIoT applications evaluated, and what metrics are most frequently reported?*

Answer: Performance evaluation in TinyML—IIoT studies combine predictive accuracy with resource-efficiency metrics. Accuracy-related measures such as classification accuracy, precision, recall, and F1-score are the most consistently reported across applications (Table 5, Table 6, Table 7, Table 8 and Table 9). These tables also show that energy consumption is highly relevant but less consistently reported. Moreover, evaluation practices vary depending on model type, hardware platform, and optimization strategy (Table 10, Table 11, Table 12, Table 13, Table 14, Table 15 and Table 16). Overall, the findings reveal a lack of standardized evaluation protocols, with performance metrics differing significantly across studies, thereby limiting direct comparisons. Future work should establish unified benchmarking frameworks that integrate accuracy, latency, memory, and energy efficiency to enable reproducible and fair evaluation of TinyML in IIoT systems.

*RQ5: What are the key limitations and open research challenges in applying TinyML to IIoT, and what future research directions are proposed?*

Answer: The review identifies several limitations in TinyML-IIoT deployments (Section 7.1). Performance evaluation is inconsistent. Accuracy is often reported, but latency, memory, and energy metrics lack standardization (Table 5, Table 6, Table 7, Table 8 and Table 9). Dataset availability is limited. Most studies use small, domain-specific datasets (Table 13 and Table 14), restricting reproducibility and generalization. Adaptive learning and multi-sensor fusion are rarely explored. Meta-learning and reinforcement learning appear in a few studies, and most systems rely on single-modality sensing. Hardware and software diversity complicates deployment (Table 12). Platforms vary widely (Table 10), and frameworks range from TensorFlow Lite Micro to custom toolchains (Table 11). Scalability and reliability remain unresolved. Most deployments are prototypes or lab-scale systems, with limited evidence of large-scale industrial integration. In summary, TinyML-IIoT research must improve evaluation standards, dataset availability, adaptive learning, sensor fusion, and cross-platform support. Future work should focus on open industrial datasets, unified benchmarking, adaptive and federated learning, multi-sensor integration, and scalable toolchains for heterogeneous hardware (Section 7.2).

### 7.2. Limitations of the Research

Although this systematic literature review followed a clear and rigorous methodology, several limitations should be acknowledged to provide a proper context for the findings.

**Search Process Limitations:** The search process relied on predefined keywords and selected scientific databases. As a result, some relevant studies may have been excluded if they used alternative terminology or were published outside the chosen sources. In addition, the large number of initial search results made exhaustive manual screening impractical, potentially leading to the omission of some relevant studies with non-explicit titles.

**Reporting Limitations In Existing Studies:** TinyML in industrial IoT is a relatively recent research area. Consequently, several published studies lack essential technical details, such as precise descriptions of the hardware platforms used, energy consumption measurements, or detailed information about the datasets. This incomplete reporting limits direct comparison across studies and reduces the reproducibility of some reported results.

**Scope and Selection Constraints:** The number of selected studies may appear limited compared to the rapid growth of research in artificial intelligence and IoT. However, this review prioritized study quality and industrial relevance over quantity. Many articles were excluded because they lacked experimental validation, provided insufficient deployment details, or repeated similar ideas without meaningful contributions.

**Heterogeneity Across Studies:** The diversity of application domains, hardware platforms, software frameworks, and evaluation metrics across the selected studies prevents quantitative comparison and meta-analysis. Despite these limitations, the reviewed studies provide a solid and representative overview of current research trends, challenges, and opportunities in TinyML-enabled industrial IoT systems. These limitations also highlight the need for better reporting practices, standardized benchmarks, and more comprehensive industrial datasets in future research.

## 8. Conclusions

This review analyzed 35 peer-reviewed studies on TinyML in IIoT systems. The studies were collected from major databases and focused on enabling ML inference on resource-constrained industrial devices. Findings were organized into application domains, hardware platforms, software frameworks, sensor modalities, datasets, model architectures, and optimization techniques. TinyML was applied to predictive maintenance, condition monitoring, anomaly detection, quality inspection, and energy management. Hardware diversity spans nRF52, STM32, and ESP32/ESP8266 boards, vision accelerators, and heterogeneous multi-processor systems. Frameworks include TensorFlow Lite Micro, Edge Impulse, custom toolchains, direct C/C++ implementations, and CMSIS-NN libraries. Sensors fall into six groups, with motion and vibration dominating, while vision, chemical, environmental, electrical, and acoustic sensors support specialized tasks. Datasets combine real-world industrial signals with public benchmarks, balancing deployment realism and reproducibility. CNNs dominate model architectures, while classical ML and MLPs remain practical for constrained devices; hybrid and recurrent models appear in niche cases. Optimization strategies rely mainly on quantization and pruning, complemented by approximate computing, meta-learning, reinforcement learning, and metaheuristics. Research gaps remain in standardized evaluation, energy and memory reporting, open industrial datasets, adaptive learning, and multi-sensor fusion. This synthesis highlights the need for efficient models, unified evaluation protocols, and realistic datasets to advance TinyML adoption in industrial environments.

## Figures and Tables

**Table 1:** Summary of existing systematic literature reviews: focus areas and key limitations.

Ref.	Year	Focus	Main Limitations
**TinyML-focused Reviews**			
[23]	2022	Systematic review of TinyML for low-power AI and IoT; focused on model compression and quantization.	Did not include industrial or IIoT use cases; focused on general IoT.
[24]	2024	Broad survey on TinyML; covered algorithm, hardware, and co-design approaches.	Lacked analysis of industrial integration and IIoT applications.
[19]	2024	Review of TinyML for predictive maintenance using vibration and temperature sensors.	Focused only on predictive maintenance; ignored broader IIoT areas.
**Reviews Focused on ML in IIoT**			
[25]	2023	Mapping of ML-based anomaly detection in IIoT; surveyed industrial ML approaches.	Depended on cloud/server ML; no TinyML or edge inference.
[26]	2023	Review of predictive maintenance techniques for Industry 4.0.	Ignored TinyML and energy-efficient on-device ML.
[27]	2024	Review of ML on IoT manufacturing data; emphasized model interpretability.	Did not address TinyML or embedded system constraints.
[28]	2023	Systematic review and taxonomy of ML in smart manufacturing.	Focused on general ML; lacked TinyML or low-power edge systems.
**Surveys on TinyML and Edge Intelligence**			
[29]	2025	TinyML, focused mainly on general IoT and embedded AI scenarios.	Did not specifically analyze TinyML deployment in IIoT environments.
[30]	2026	Review of TinyML in IIoT within the transition from Industry 4.0 to Industry 5.0.	Emphasized conceptual perspectives of Industry 5.0 rather than detailed analysis of TinyML deployment.
**This Study**			
**This Study**	2026	TinyML; IIoT, model optimization, hardware components, software frameworks, datasets.	Broader Edge AI frameworks and large-scale industrial AI systems are beyond the scope.

**Table 2:** Search terms and number of retrieved records across selected databases.

No.	Search Term	IEEE	Elsevier	Springer	ACM
Main Keywords					
1	Edge AI	13,917	214,725	15,007	77,740
2	TinyML	721	383	1	346
3	Industrial Internet of Things (IIoT)	4484	4814	31	802
4	Tiny Machine Learning	1681	27,963	4366	10,960
5	On-device Machine Learning	1116	220,693	20,762	85,916
6	Micro ML	1044	813,641	55,166	23,755
7	Embedded Machine Learning	33,800	184,910	22,622	90,328
8	Low-power ML	868	1,000,000+	96,996	105,998
9	Industry 4.0	12,347	363,694	18,096	19,677
10	Smart Factory	6669	36,374	2150	6142
Combined Keywords (Main Term + Domain/Application)					
11	Edge AI AND IIoT	203	2165	351	395
12	TinyML AND IIoT	7	44	7	34
13	TinyML AND Industrial IoT	35	175	33	121
14	TinyML AND Smart Factory	6	44	7	71
15	TinyML AND Predictive Maintenance	23	144	34	194
16	TinyML AND Anomaly Detection	87	176	41	129

## Data Availability

No new data were created or analyzed in this study. Data sharing is not applicable to this article.
